# Genomic analysis of the Ixworth chicken: insights into a local dual-purpose breed

**DOI:** 10.1186/s12864-026-12732-9

**Published:** 2026-03-11

**Authors:** Hendrik Bertram, Muhammad Jawad, Susann Michanski, Inga Tiemann, Armin O. Schmitt, Mehmet Gültas

**Affiliations:** 1 Statistics and Data Science Group, Department of Agriculture, South Westphalia University of Applied Sciences, Lübecker Ring 2, Soest, 59494 North Rhine-Westphalia Germany; 2 Breeding Informatics Group, Department of Animal Sciences, Georg August University, Margarethe von Wrangell-Weg 7, Göttingen, 37075 Lower Saxony Germany; 3 Center for Integrated Breeding Research (CiBreed), Georg August University, Von-Siebold-Str. 8, Göttingen, 37075 Lower Saxony Germany; 4 Precision Livestock Farming, Faculty of Agricultural Sciences and Landscape Architecture, Osnabrück University of Applied Sciences, Am Krümpel 31, Osnabrück, Lower Saxony 49090 Germany

**Keywords:** Ixworth, Whole-genome sequencing, Dual-purpose, Selection signature, Biodiversity, Inbreeding

## Abstract

**Background:**

Intensive genetic selection in commercial poultry has greatly enhanced productivity, but it has also caused substantial genetic erosion, affecting long-term sustainability. Body weight and growth rate as well as egg production are considered antagonistic traits, leading to highly specialized broiler and layer lines with further loss of diversity. Local breeds, such as the British Ixworth dual-purpose chicken, are vital reservoirs of biodiversity, yet the genomic basis of their balanced traits remains poorly understood. At the same time, ethical concerns regarding male chick culling have led several European countries to ban the practice in recent years, renewing interest in dual-purpose systems in which both sexes can be viably utilized. By addressing both genetic erosion and chick culling, dual-purpose chickens can contribute to more sustainable poultry production.

**Results:**

To investigate the genetic architecture of the Ixworth chicken, we conducted high-resolution whole-genome sequencing, and compared it with its wild progenitor and specialized commercial lines for layers and broilers. Our analyses identify the Ixworth chicken as genetically distinct, with moderate nucleotide diversity, low inbreeding, and rapid decay of linkage disequilibrium, suggesting limited historical selection despite possible recent bottlenecks associated with its critical status. Its genetic architecture further revealed distinguished selection signatures balancing meat productivity (chromosomes 1, 6, 28) with egg-laying performance (chromosomes 1, 3, 10), contrasting with the narrow selection for growth in broilers and reproductive efficiency in layers. Notably, we discovered a distinct selective sweep on chromosome 4 (90.10–90.30 Mb) that harbors the developmental regulator *CYP26B1*, the exocytosis regulator *EXOC6B*, regulatory long non-coding RNAs, and co-localizes with QTLs for cooking loss and fatty acid composition. This alignment provides a compelling genomic basis for the breed’s historical reputation for superior table quality, a trait likely selected either deliberately or as a fortunate consequence of selection on a linked developmental pathway.

**Conclusions:**

This study uncovers the complex genetic legacy of the British Ixworth dual-purpose breed, offering insights into population parameters and positive selection signatures that may help to enhance biodiversity and animal welfare in future breeding programs and guide conservation efforts of the breed.

**Supplementary Information:**

The online version contains supplementary material available at 10.1186/s12864-026-12732-9.

## Introduction

Intensive genetic selection for commercial broiler and layer chickens has resulted in remarkable gains in productivity, but this unidirectional specialization has come at a significant cost. As a deliberate consequence of breeding programs, commercial lines tend to become genetically homogeneous, exhibiting reduced genetic diversity [1], increased disease susceptibility [2], and compromised animal welfare in both broilers and layers [3, 4]. Growing ethical concerns regarding welfare within poultry production systems are increasingly prominent, especially in light of recent legislative changes such as the prohibition on culling of male layer chicks in European countries like Germany [5], Austria [6], and France [7]. The ban highlights the urgent need for feasible alternatives, as rearing male layer chicks remains economically unsustainable. While in-ovo sex determination holds potential, it poses both practical and ethical challenges, as sexing accuracy improves with embryo age, requires high-throughput systems, and raises concerns about embryonic pain if selection occurs too late [8]. Consequently, the interest in dual-purpose chickens increased, as they can be used for both meat and egg production. Unlike commercial lines that require continuous reliance on specialized hatcheries, local dual-purpose breeds offer the potential for independent on-farm breeding, making them an attractive option for small-scale organic farming systems. However, the performance of dual-purpose chickens is less pronounced than those of specialized lines. For instance, Mueller et al. reported that Schweizerhuhn and Belgian Malines, two dual-purpose breeds, reached average slaughter weights of 1,317 g and 1,758 g at 9 weeks, whereas the commercial hybrid Lohmann Dual reached 2,161 g [9], and the fast-growing broiler Ross PM3 reached 2,415 g at 5 weeks [9]. Laying performance follows a similar trend. Gangnat et al. reported laying percentages of Schweizerhuhn and Belgian Malines at 62.3 % and 69.3 %, compared to 82.3 % in Lohmann Dual and 92.9 % in Lohmann Brown at 33 weeks [10]. Nevertheless, local and less intensively selected breeds remain valuable for organic systems and future breeding due to their adaptive and robust traits. Among these, the Ixworth chicken, a rare local breed, presents a compelling case study.

Developed in the 1930s by Reginald Appleyard to be an economically viable dual-purpose breed [11], the genetic background of the Ixworth chicken includes White Orpington, White Sussex, White Minorca, Jubilee and Indian Game, with phylogenetic analyses confirming its similarity to other British dual-purpose and layer breeds [12]. In addition to serving as a potential solution to unethical culling, this breed shows improved welfare indicators, including low mortality and high nest acceptance [11]. Its genetic diversity may also enhance resilience to diseases and behavioral stressors, improving overall welfare. Hence, the Ixworth chicken represents a valuable reservoir of untapped genetic diversity that is potentially critical for countering increasing homozygosity in commercial lines. It can further serve as a capacity for future adaptation to enable responses to unforeseen environmental changes without impairing fitness and productivity.

The purebred status of the Ixworth chicken provides an opportunity to explore the genomic consequences of balanced dual-purpose breeding in contrast to the unidirectional selection typical of specialized lines. Despite this potential, the Ixworth remains critically endangered and insufficiently characterized, with limited information available on its genetic background and performance traits. To enhance both awareness and understanding of this breed, we conducted a comprehensive whole-genome characterization aimed at closing the existing knowledge gap. We tested the following research hypotheses: (i) its dual-purpose history has left selection signals at loci affecting growth and reproductive traits, and (ii) its less-intensive selection has preserved higher genome-wide diversity than in commercial broiler and layer lines. Using whole-genome resequencing data, we compared Ixworth chickens with commercial broilers, commercial layers, and red junglefowl as a proxy for the primary wild progenitor of domestic chickens [13], assessing population genetic parameters and signatures of selection.

## Material and methods

### Ixworth population

The Ixworth chickens sampled in this study were maintained at the Campus Frankenforst of the Faculty of Agricultural, Nutritional and Engineering Sciences of the University of Bonn, Germany. At the time of sampling, the closed population consisted of roughly 1,000 Ixworth chickens. Over six generations, breeding groups were established, each comprising on average 70 hens and 10 roosters. Breeding candidates were selected based on morphological traits, laying performance of the dam, and body weight of the sire. Selection was applied to approximately 155 hatched chicks per generation. Mating was random with respect to roosters, while egg production was recorded per hen using individual trap nests.

Since the Ixworth chicken is a local breed, data on performance traits is limited. However, Becker et al. [11] began data collection on this population, and readers are referred to their work for a full description and comparisons of performance with regard to other chicken breeds. In terms of growth, Ixworth cockerels average 2344 g body weight at 12 weeks on a cockerel-layer diet, with a mortality rate of 1–2 %. Daily weight gain averages 27.4 g, and the feed conversion ratio is estimated at 2.48. Carcass composition averages 31.8 % leg meat, 17.5 % breast meat, and 12.5 % wing meat, with leg and breast yield negatively correlated. For laying performance, Ixworth hens average 195 eggs (56 g each) in 52 weeks, with nest acceptance exceeding 97.5 %. The first egg is typically laid at 139 days, and by 72 weeks, hens average over 3 kg.

### Sample extraction and sequencing

Liver samples of 50 male Ixworth chickens were collected directly following regularly scheduled slaughter and immediately snap-frozen in liquid nitrogen and stored at −80°C until further processing. Genomic DNA was extracted from approximately 20 mg slices of each liver sample using the peqGold Blood and Tissue DNA Mini Kit (VWR, Darmstadt, Germany), following the manufacturer’s protocol. The protocol included an RNase A digestion step to reduce the impact of RNA contamination. The quality and quantity of the isolated genomic DNA were assessed using a NanoDrop$^\text {TM}$ 2000 spectrophotometer at 260/280 nm ratio (Thermo Fisher Scientific, Wilmington, DE, USA). Following normalization, the genomic DNA samples were adjusted to a final volume of 50 $\mu$L and submitted for whole-genome sequencing (WGS) to the Competence Centre for Genomic Analysis (CCGA) in Kiel, Germany. Following further DNA quality controls during library preparation, one sample was discarded, yielding 49 samples remaining for WGS. Sequencing libraries were constructed at the CCGA, and WGS was performed using Illumina NovaSeq 6000 to generate 2 x 151 bp paired-end reads, aiming for an average read depth of approximately 26x to ensure robust coverage. On average, this yielded 182.8 million reads per Ixworth chicken sample. Raw reads from the WGS data were uploaded to the European Nucleotide Archive (ENA) under accession number *PRJEB89160*.

### Public data sources

Sequences of broilers (two commercial lines), layers (commercial white and brown layers), and a red junglefowl population [1] were retrieved from the ENA project *PRJEB30270* using enaBrowserTools [14]. Briefly, red jungle fowl consisted of 25 DNA samples collected in 1999 from a population in northern Thailand, initially caught in 1997 and maintained with random mating. The broiler group includes two sire lines, referred to as line A (Indian River International, established in 1980) and line B (French origin, developed in 1970), kept in large population sizes (>10,000) with 20 DNA samples each. The layer group consists of 25 birds each from white and brown egg-laying parental lines of unknown population sizes, representing parental lines of Lohmann Breeders GmbH, Germany, derived from White Leghorn and Rhode Island Red [1]. While both layer lines are purebred, the status of the broiler lines is not explicitly described [1]; however, it is highly likely that they are also purebred given their status as foundational sires. At the time of the study, three layer samples were not retrievable. An overview of the different populations under study is shown in Table 1.

**Table 1:** Sampling information of the chicken populations used in this study

Population	n	Origin
Ixworth	49	Germany
Red junglefowl	25	Thailand
Broiler line A	20	Indian River International (Texas), USA
Broiler line B	20	France
Brown layer	23	Lohmann Breeders GmbH, Germany
White layer	24	Lohmann Breeders GmbH, Germany

### Alignment and variant calling

Variant calling was conducted using a Snakemake [15] pipeline based on the Genome Analysis Toolkit (GATK) version 4.6.1.0 [16], which we adapted from Köster et al. [17], to enable reproducibility.

At first, raw paired-end sequencing reads were preprocessed and cleaned using fastp (version 0.23.4) [18] to obtain high quality reads, decreasing false positives in subsequent aligning and variant calling. This process involved the automatic detection and removal of adapter sequences (--detect_adapter_for_pe), trimming of poly-G tails (--trim_poly_g), and removal of low-quality bases (mean quality scores below 20) from the 3’-end using a sliding window approach (--cut_tail,--cut_mean_quality 20). Reads shorter than 50 bp (--length_required 50) or with more than 30 % of bases falling below a Phred quality score of 20 were discarded (--unqualified_percent_limit 30). This initial filtering step removed an average of 5.6 % of reads from the Ixworth chicken dataset and 7.2 % from the public datasets due to adapter contamination or low quality, mostly found at the tails. Pre-alignment duplication rates calculated by fastp were low, averaging 2.8 % for Ixworth chicken and 0.6 % for the public data.

The resulting high-quality reads were aligned to the chicken reference genome for red junglefowl, GRCg6a (Ensembl release 112) [19], using the BWA-MEM algorithm (version 0.7.18) [20] with default parameters. Mean alignment rates ranged from 99.3 % to 99.4 % across all samples. The red junglefowl reference was chosen to provide an appropriate ancestral background, required for comparing the diverse domestic populations under study. Following alignment, BAM files were sorted and indexed using SAMtools (version 1.21) [21], and PCR duplicates were marked for exclusion with Picard (version 3.3.0) [22]. Duplicate reads constituted 10.2 % of reads in the deeper-coverage Ixworth chicken samples and 1.0 % in the other populations. The resulting mean genome coverage was 25.4x for the Ixworth chicken population and 9.9x for the other populations, with over 97 % of the genome covered by at least one read across all samples. Quality control metrics from fastp, FastQC (version 0.12.1) [23], and Qualimap (version 2.3) were aggregated and visualized using MultiQC (version 1.25.1) [24] to ensure data integrity at each stage.

Following GATK best practices, we performed quality score recalibration (BQSR) using the BaseRecalibrator and ApplyBQSR commands, with known variant sites from Ensembl (release 112) used as a reference. Per-sample genotype likelihoods were generated with HaplotypeCaller in GVCF mode. The individual GVCF files were combined with CombineGVCFs and jointly genotyped across all 161 samples using GenotypeGVCFs, yielding a raw multisample VCF file containing 27.96 million variants, which included 3.4 million indels and 1.76 million multi-allelic sites. The raw VCF file was subjected to a filtering protocol to generate a high-confidence SNP dataset. First, we applied GATK’s recommended hard filters for SNPs to remove potential false positives (QD<2.0, QUAL<30.0, SOR>3.0, FS>60.0, MQ<40.0, MQRankSum<−12.5, ReadPosRankSum<−8.0). Afterwards, indels and multi-allelic sites were excluded using BCFtools (version 1.21) [25]. For all downstream population genetic analyses, the dataset was further filtered to include only bi-allelic SNPs located on autosomes. We retained only variants with a genotyping rate of at least 50 % across all individuals and a minor allele count of at least 2 copies. These filters reduced low-quality and singleton calls and avoided sex-chromosome effects. The final dataset consisted of 17.99 million high-quality autosomal SNPs, with a mean genotype call rate of 98.29 %. Of these, 11.59 million SNPs were found in the Ixworth chicken population, of which 510,967 were private (polymorphic only within Ixworth). More detailed alignment and variant calling statistics are provided in Supplementary Table S1.

### Population statistics

All statistical analyses, data wrangling, and data visualizations were performed in R (version 4.4.1) [26] using the tidyverse (version 2.0.0) [27] package. We calculated each of the following metrics separately for each population based on the final SNP dataset.

To investigate the genetic relationships among the chicken populations, we first performed principal component analysis (PCA). To minimize artifacts from linkage disequilibrium (LD), the SNP set was pruned using PLINK (version 1.9) [28]. This was achieved by removing SNPs in sliding windows of 50 SNPs with a step size of 5 SNPs that exhibited pairwise LD ($r^2$) greater than 0.2 (--indep-pairwise 50 5 0.2). The PCA was then conducted on this pruned dataset using the SNPRelate R package (version 1.40.0) [29].

To infer ancestral population components and quantify admixture proportions, we used ADMIXTURE (version 1.3) [30]. The ADMIXTURE analyses were run on the LD-pruned SNP set with 20-fold cross-validation (--cv=20) across k-values ranging from $k = 1$ to $k=10$ ancestral populations. The optimal k was defined as the value with the lowest mean cross-validation error.

To assess genetic variation within each population, we calculated several commonly used diversity indices. Nucleotide diversity ($\pi$) was estimated in sliding windows of 40 kb with a 20 kb step size using VCFtools (version 0.1.16) [31]. Additionally, VCFtools was used to calculate individual observed heterozygosity ($H_o$), expected heterozygosity ($H_e$), and the method-of-moments inbreeding coefficient (*F*). To specifically quantify recent and ancient inbreeding, we identified runs of homozygosity (ROH) using the hidden Markov model implemented in BCFtools (version 1.21) [32]. We restricted ROH results to homozygous segments of at least 300 kb containing a minimum of 10 SNPs. For comparative purposes, individual ROH were binned by length (0.3–1.3, 1–2, 2–4, 4–8, 8–10, 10–16, and >16 Mb), following Talebi et al. [33], who previously studied the same public dataset on layers, broilers, and red junglefowl. The genomic inbreeding coefficient ($F_{ROH}$) was then calculated for each individual as the total length of all ROH segments divided by the total autosomal genome size, estimated to be 960.8 Mb.

Genetic differentiation between all pairs of populations was quantified using the Weir and Cockerham fixation index ($F_{ST}$) [34], calculated with VCFtools in 40 kb windows with a 20 kb step size.

To make inferences about recent historical effective population size ($N_e$) and demographic events like bottlenecks or selection, we assessed the rate of LD decay for each population. A more rapid decay of LD might be indicative of a larger historical $N_e$, whereas slower decay could suggest a greater impact from past demographic events. Hence, we estimated $r^2$ for each population between all SNPs within a 500 kb distance using PopLDdecay (version 3.43) [35]. To smooth the visualization while preserving extreme values, we binned the mean pairwise $r^2$ estimates into 1 kb windows, starting after the first 1 kb.

To identify genomic regions shaped by positive selection, we employed two complementary haplotype-based statistics after phasing and imputing the SNP dataset with Beagle (version 5.4) [36] using parameters iterations=50 and burnin=20. First, to detect signatures of ongoing or incomplete selection within the Ixworth chicken population, we calculated the integrated haplotype score (iHS). This statistic identifies genomic regions where haplotypes carrying either the ancestral or derived allele have swept to high frequency faster than expected under neutrality. Second, to detect loci under differential selection close to fixation between populations, we used the cross-population extended haplotype homozygosity (XP-EHH) statistic. This was performed in pairwise comparisons between the Ixworth chicken population and each of the other populations (layers, broilers, and red junglefowl). Herein, we analyzed the absolute XP-EHH and iHS scores to identify strong selective pressures regardless of which population or allele was the target of selection. This allows for the discovery of not only regions selected in Ixworth chickens but also regions selected in the commercial lines that may represent valuable targets for future breeding programs. Both iHS and XP-EHH statistics were calculated using the rehh R package (version 3.2.2) [37], with the red junglefowl reference allele designated as ancestral. To identify strong regions under selection while reducing SNP-level noise, the absolute standardized scores were averaged in sliding windows of 40 kb with a 20 kb step size, requiring at least 10 SNPs per window. These are denoted as $|XP-EHH|_w$ and $|iHS|_w$. Windows falling within the top 0.1 % of the empirical distribution for each respective comparison were considered candidate regions under strong positive selection, which is a heuristic approach similar to previous studies on selection [38–40]. Finally, adjacent candidate regions were merged to define broader putative selective sweeps for functional annotation. To understand the biological importance of the identified selective sweeps, candidate regions were functionally annotated by genes retrieved from Ensembl gene annotations (release 112). For genes with missing annotations, functional information was inferred by performing a BLAST search [41] of the gene sequence against the NCBI ClusteredNR database [42], limited to birds. Finally, all candidate regions were cross-referenced with the AnimalQTLdb database (release 55) [43] to identify overlaps with previously reported Quantitative Trait Loci (QTLs) in chickens.

## Results

### The Ixworth chicken breed is genetically distinct

**Fig. 1:**
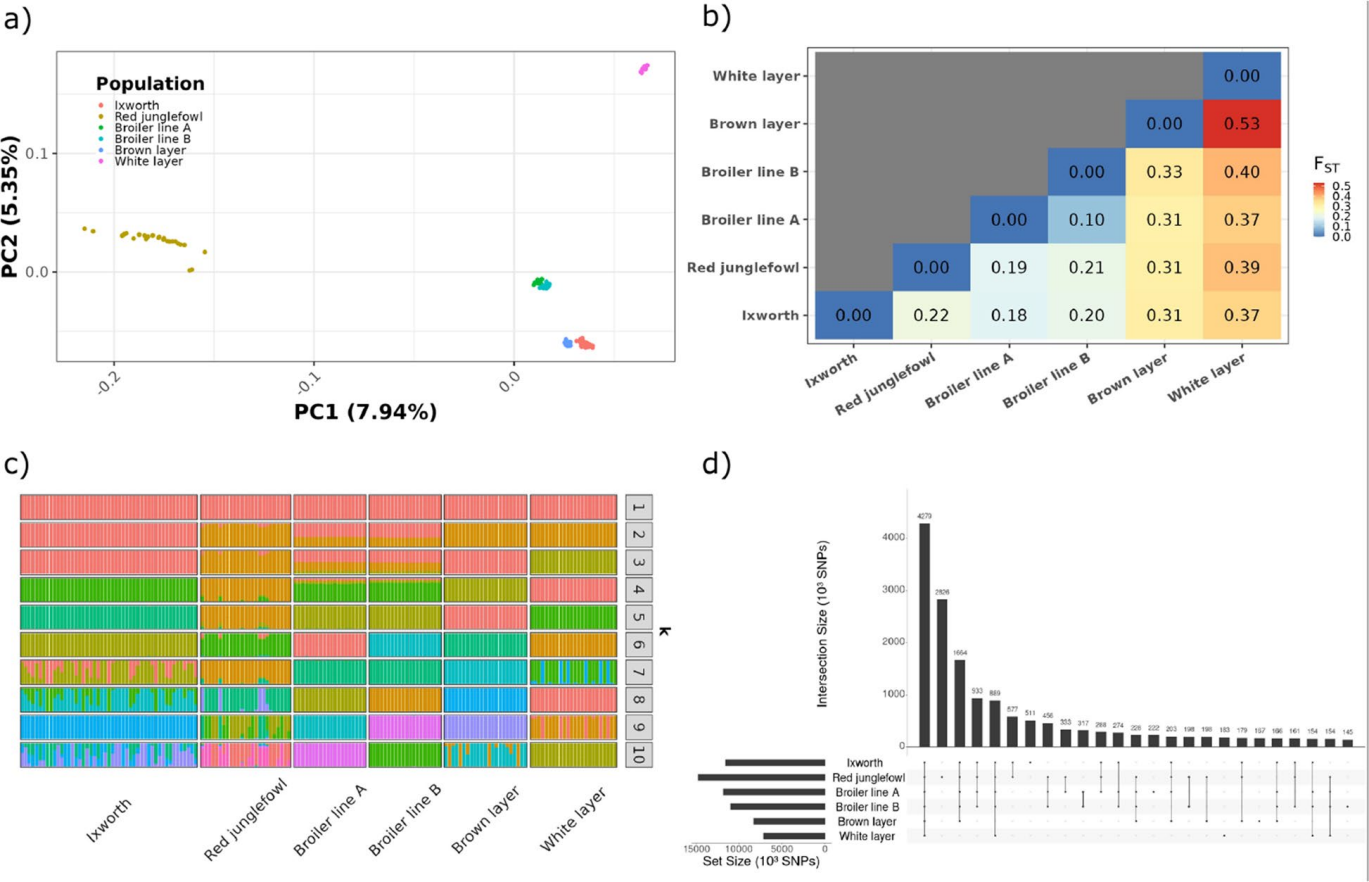
Genetic structure analysis of chicken populations of Ixworth, red junglefowl, two broiler lines (A and B), and brown as well as white layer lines. **a** Principal component analysis of the chicken populations. **b** Genetic differentiation analysis between populations using genome-wide average weighted $F_{ST}$. **c** Admixture analysis between populations for $k = 1$ to $k = 10$ clusters, where $k = 6$ shows the lowest cross-validation error. **d** Distribution of shared SNP frequencies between populations

To investigate the genetic relationships among the Ixworth chicken, commercial, and red junglefowl populations, we first performed PCA. The first two principal components (PCs) of the PCA, explaining 13.29 % of the total genetic variation, revealed four distinct and well-separated clusters corresponding to i) red junglefowl, ii) both broiler lines, iii) white layers, and iv) brown layers and Ixworth chickens, as shown in Fig. 1a. The red junglefowl cluster was the most dispersed, whereas the cluster of both broiler lines was the most compact. The brown and white layer lines were strongly separated. PC1 primarily separated the red junglefowl from the domestic populations, while PC2 distinguished the white layer line from the other clusters. Contrary to an expected intermediate position between broilers and layers, the Ixworth chicken population is found closer to the brown layer population than to the broiler populations. However, these trends are not fully observed for PCs three to five, which explain an additional 13.27 % of the total variance (Supplementary Figure S1).

We further quantified these genetic separations by calculating the genome-wide pairwise $F_{ST}$. The $F_{ST}$ values confirmed some of the visual clustering, with moderate to high levels of differentiation among all populations (Fig. 1b). Differentiation was lowest between the two broiler lines ($F_{ST} = 0.10$) and highest between the two layer lines ($F_{ST} = 0.53$). Interestingly, the Ixworth chicken population showed moderate differentiation from broilers ($F_{ST} = 0.18$ for line A, $F_{ST} = 0.20$ for line B) and red junglefowl ($F_{ST} = 0.22$), and higher differentiation from the layer lines ($F_{ST} = 0.31$ for brown, $F_{ST} = 0.37$ for white), in contrast to the PCA-clustering of Ixworth chickens and brown layers (Fig. 1a). Analysis of individual chromosomes revealed that the highest differentiation between Ixworth chicken and the other populations consistently occurred on chromosome 24 (Supplementary Figure S2), while microchromosomes, particularly chromosome 16, typically showed lower levels of differentiation.

Admixture analysis supported the genetic integrity of the Ixworth chicken breed. The lowest cross-validation error was observed at $k=6$, which assigned distinct ancestral components to all populations (Fig. 1c). Cross-validation errors at increasing k-values are shown in Supplementary Figure S3. Hence, all populations appeared largely without admixture, with only minor shared ancestry components observed between the modern domestic populations and the red junglefowl. At lower values of k ($k < 6$), broader ancestral relationships were observed, highlighting the close relationship between the broiler populations and a potentially closer domestication history between Ixworth chickens and broilers ($k = 4$) as well as Ixworth chickens and the brown layer ($k = 3$). At higher values of k ($k> 6$), potential substructures within the Ixworth chicken population could be observed.

Finally, we analyzed the distribution of shared and private SNPs across populations (Fig. 1d). While a large core set of 4.28 million SNPs (23.8 %) was shared among all populations, the Ixworth chicken population possessed 510,967 private SNPs, a number surpassed only by the red junglefowl (2.83 million private SNPs). Pairwise comparisons showed that the Ixworth chicken shares the highest proportion of its SNPs with the red junglefowl (84.5 %), and the lowest with the white layer line (53.0 %).

### The Ixworth chicken breed harbors high genetic diversity and low inbreeding

The populations exhibited substantial differences in overall genetic diversity, which we quantified by assessing the number of polymorphic sites and the nucleotide diversity index $\pi$ to test the hypothesis that the Ixworth chicken breed has retained high genetic diversity (Table 2). The red junglefowl contained the highest number of polymorphic sites (14.77 million), followed by the Ixworth chicken (11.59 million) and broiler populations (10.99–11.80 million). The layer lines showed a strong reduction in variation, with the fewest polymorphic sites (7.15–8.30 million). This is emphasized by $\pi$, where red junglefowl also displayed the highest value ($\pi = 0.41\%$), followed by the Ixworth chicken ($\pi = 0.35\%$) and broilers ($\pi$ between 0.33 % and 0.36 %), and finally the layer lines ($\pi$ between 0.18 % and 0.22 %), which exhibited the lowest diversity. Similar to the findings regarding $F_{ST}$, $\pi$ was typically lowest in microchromosomes, such as chromosomes 16, 22, or 29 to 32 (Supplementary Figure S4). Furthermore, Ixworth chickens also exhibited higher levels of heterozygous sites. Although $H_e$ was similar across all populations, with red junglefowl having slightly lower values, the Ixworth chicken population had the highest $H_o$ value (0.31) relative to its $H_e$ (0.30) among all populations (Table 2).

**Table 2:** Summary statistics for each chicken population. Means and standard deviations were calculated from individual estimates obtained for each chicken within each population. Columns include total number of SNPs ($n_{\textrm{SNP}}$, millions), observed heterozygosity ($H_o$), expected heterozygosity ($H_e$), method-of-moments inbreeding (*F*), ROH-based inbreeding ($F_{ROH}$), and nucleotide diversity ($\pi$, percentage, 40 kb windows)

Population	$n_{SNP}$ (M)	$H_o$	$H_e$	*F*	$F_{ROH}$	$\pi$ (%)
Ixworth	11.59	0.31 ± 0.02	0.30	−0.02 ± 0.06	0.17 ± 0.04	0.35 ± 0.15
Red junglefowl	14.77	0.26 ± 0.03	0.28	0.05 ± 0.13	0.12 ± 0.10	0.41 ± 0.16
Broiler line A	11.80	0.30 ± 0.02	0.31	0.04 ± 0.07	0.14 ± 0.05	0.36 ± 0.16
Broiler line B	10.99	0.31 ± 0.00	0.32	0.03 ± 0.03	0.16 ± 0.02	0.33 ± 0.15
Brown layer	8.30	0.28 ± 0.03	0.32	0.13 ± 0.10	0.38 ± 0.04	0.22 ± 0.12
White layer	7.15	0.29 ± 0.02	0.31	0.08 ± 0.08	0.43 ± 0.03	0.18 ± 0.12

We next assessed inbreeding using two genotype-based methods. The method-of-moments inbreeding coefficient was lowest in the Ixworth chicken ($F = -0.02$), while the inbreeding coefficient based on ROH placed the Ixworth chicken ($F_{ROH} = 0.17$) slightly above red junglefowl ($F_{ROH} = 0.12$) and the two broiler lines ($F_{ROH}$ between 0.14 and 0.16) but substantially lower than the layer lines, exhibiting $F_{ROH}$ above 0.38 (Table 2). Conformity of *F* and $F_{ROH}$ is shown in Supplementary File S1.

The analysis of ROH further presented the recent inbreeding history (Table 3). The layer lines showed the greatest burden of homozygosity, with a mean total ROH length of 366.83 Mb (brown) and 415.80 Mb (white). This was driven by an overall high number of ROH segments across all lengths below 8 Mb, with a mean of 427.39 and 512.54 ROH segments per individual, respectively. In contrast, the Ixworth chicken and broiler populations had much lower mean total ROH lengths (158.99 Mb and 137.27–154.26 Mb, respectively) and fewer long ROH segments above 4 Mb. While no individual in the Ixworth chicken population carried a ROH segment longer than 8 Mb, we observed individuals carrying such long ROH segments in broiler line A, brown layers, and red junglefowl. The red junglefowl had the lowest mean total length of ROH (116.15 Mb), but also the highest variance among individuals, where a single individual had a ROH segment of length 9.93 Mb. Overall, only few ROH segments above 8 Mb were detected across any population.

Accordingly, based on SNP markers, the Ixworth chicken population contained a similar number of polymorphic sites (11.59 million) as the broilers (10.99–11.80 million) while the layers showed a strongly reduced number of polymorphic sites (7.15–8.30 million) and red junglefowl an increased number (Table 2).

**Table 3:** Overview of ROH segments within chicken populations. $^1$ Mean number of ROH segments per individual of a population divided into length classes. $^2$ Minimum, maximum, and mean length of ROH segments per population. $^3$ Total length of all ROH segments per population. $^4$ Mean and standard deviation of the total length of all ROH segments per individual of a population. $^5$ Mean number of ROH segments per individual

Metric	Ixworth	Red junglefowl	Broiler line A	Broiler line B	Brown layer	White layer
0.3-1Mb$^1$	182.37	85.00	149.65	177.75	315.17	389.04
1-2Mb$^1$	34.83	29.96	29.85	34.15	86.39	98.13
2-4Mb$^1$	5.61	8.84	6.10	5.25	23.22	24.13
4-8Mb$^1$	0.16	0.80	0.35	0.25	2.57	1.25
8-10Mb$^1$	0.00	0.04	0.05	0.00	0.00	0.00
10-16Mb$^1$	0.00	0.00	0.00	0.00	0.04	0.00
>16Mb$^1$	0.00	0.00	0.00	0.00	0.00	0.00
Min. L. (Mb)$^2$	0.30	0.30	0.30	0.30	0.30	0.30
Max. L. (Mb)$^2$	5.48	9.93	8.56	5.03	10.42	6.42
Mean L. (Mb)$^2$	0.71	0.93	0.74	0.71	0.86	0.81
Total L. (Mb)$^3$	7790.56	2903.82	2745.33	3085.30	8437.14	9979.13
Mean Total L.$^4$ (Mb)	158.99	116.15	137.27	154.26	366.83	415.80
SD Total L.$^4$	35.12	95.21	48.40	18.56	39.96	23.61
Mean N.$^5$	222.98	124.64	186.00	217.40	427.39	512.54

### Distinct patterns of linkage disequilibrium decay across chicken populations

**Fig. 2:**
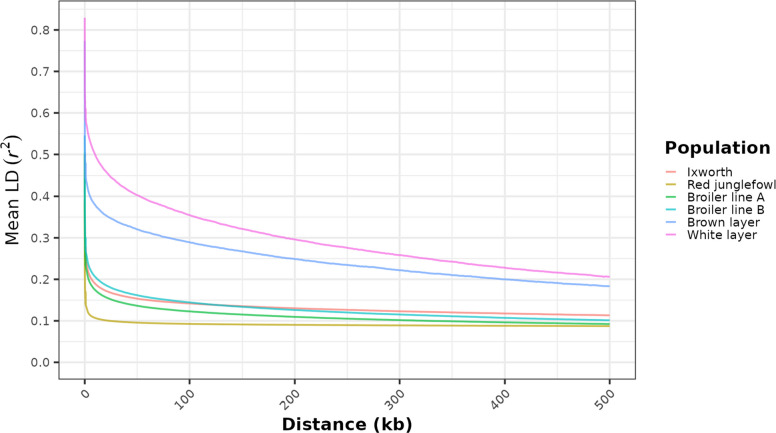
Estimated LD decay across chicken populations. Mean LD ($r^2$) between SNP pairs is plotted against physical distance. Distances less than 1 kb are shown at full resolution, and distances greater than 1 kb are averaged in 1 kb bins to smooth the visualization

To assess historical $N_e$ and selection intensity, we analyzed the decay of LD across a 500 kb distance for each population (Fig. 2). The analysis revealed relevant differences in LD patterns, separating the highly selected layer lines from the other populations. The commercial layers exhibited both the highest initial LD and the slowest rate of decay. At short distances, their average $r^2$ values reached 0.82 and 0.74, respectively, and remained elevated across long physical distances between loci. Conversely, LD decayed rapidly in the other populations. The red junglefowl population showed the most rapid decay and the lowest overall LD, with $r^2$ reaching a maximum of only 0.43 at short distances. The Ixworth chicken and broiler populations displayed intermediate trends, with rapid initial LD decay and maximum $r^2$ values between 0.54 and 0.58. The layer lines maintained the highest background LD, plateauing at $r^2$ values of approximately 0.18 (white) and 0.16 (brown). The red junglefowl population and broiler lines converged to the lowest background LD ($r^2 \approx 0.09$), while the Ixworth chicken population stabilized at a slightly higher level of $r^2 \approx 0.11$. Chromosome-specific LD decay plots for each population are provided in Supplementary File S2.

### Genomic signatures of selection reflect divergent production goals

#### Selection in Ixworth chicken targets genes related to balanced growth and reproduction

**Fig. 3:**
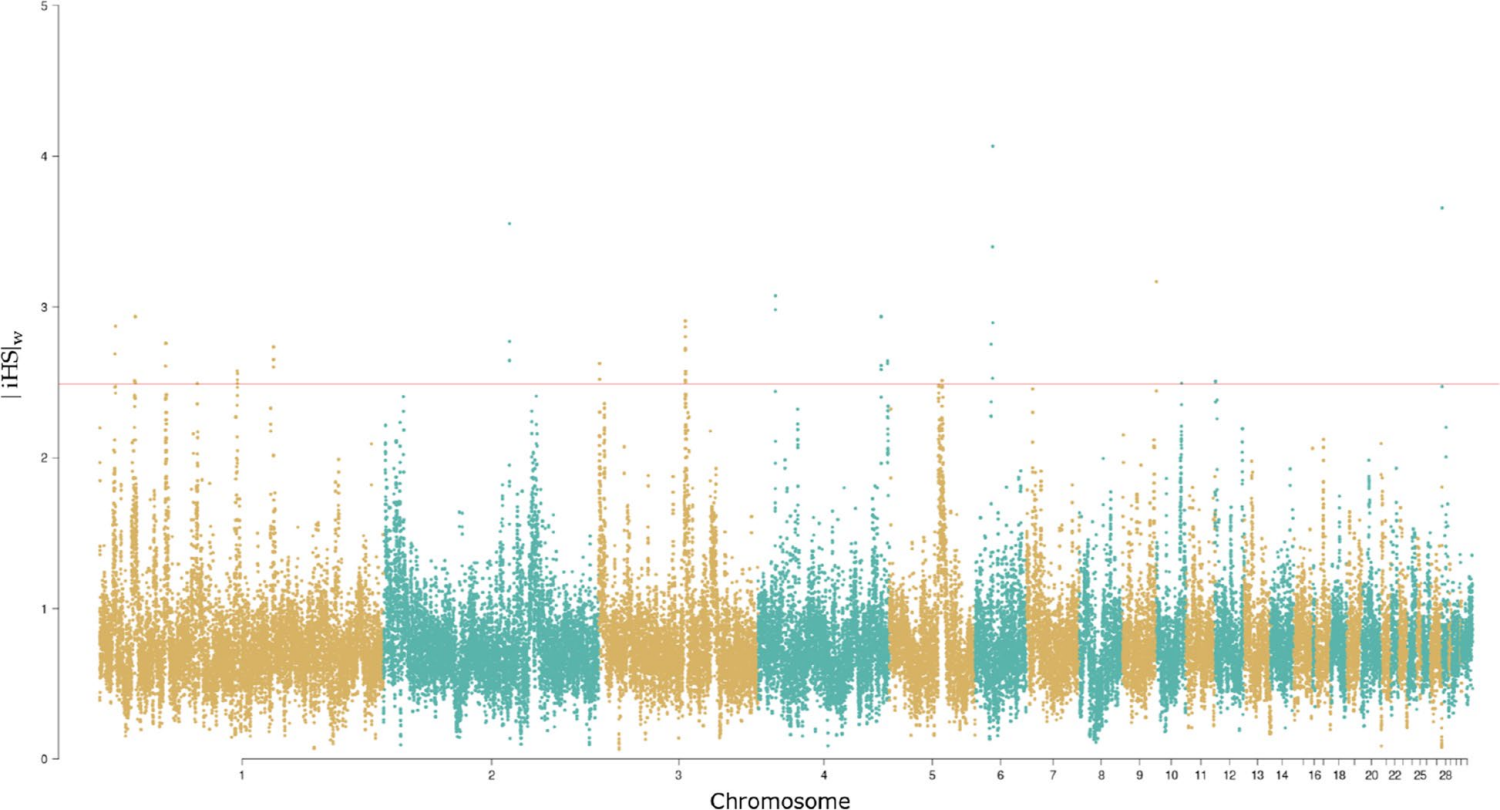
Distribution of aggregated means of the integrated haplotype score ($|iHS|_w$) within the Ixworth chicken population for 40 kb windows across the whole genome. Strong selective sweeps (top 0.1 %, $|iHS|_w> 2.49$) are indicated by the red line

Our genome-wide scan for selection within the Ixworth chicken population identified 25 non-overlapping regions under strong positive selection (top 0.1 % of windows, $|iHS|_w> 2.49$). These candidate regions based on $|iHS|_w$, distributed across chromosomes 1, 2, 3, 4, 5, 6, 9, 10, 12, and 28 (Fig. 3), contained genes associated with production, immunity, and behavior (Supplementary Table S2 for a complete list). A list of key candidate genes and their putative functions is provided in Table 4. These extreme regions ranged from $|iHS|_w$ of 2.49 to 3.66 with a mean of 2.77 and their distribution and direction are shown in Supplementary File S3.

**Table 4:** Candidate regions and genes of interest under positive selection within the Ixworth population according to $|iHS|_w$. Annotations for gene symbols marked by asterisks (“**”) were annotated by BLAST

Location (Mb)	Genes and functions
chr1:10.70–10.74	CACNA2D1: part of the voltage-dependent calcium channel complex; role in embryonic development [44]
chr1:24.76–24.82	CFTR: role in mucosal immunity; member of ABC transporters which functions as chloride channel [45]
	ASZ1: involved in piRNA pathway and transposon silencing; role in fertility [46]; candidate gene associated with growth and meat productivity [47]
chr1:45.80–45.84	ELK3: ETS transcription factor; candidate gene associated with feed conversion ratio [48]
	CDK17: cyclin dependent kinase; upregulated in Th1-like T cells; potential role in chicken antiviral immunity [49]
chr1:46.06–46.10	NEDD1: role in centrosome recruitment of $\gamma$-tubulin; essential for microtubule nucleation [50]; candidate region associated with feather pecking [51]
chr1:67.94–67.98	DAG1*: dystroglycan; hub gene correlated with muscle development [52]
	INTS13: role in development and transcription factor recruitment [53]; candidate gene associated with egg weight [54]
chr3:60.22–60.36 & chr3:60.40–60.46	RNF217*: E3 ubiquitin-protein ligase; potential role in immunity; candidate gene related to heat stress [55]
	NKAIN2: transmembrane protein; candidate gene associated with age at first egg [56]
chr4:12.06–12.12	CDX1/CDX4B*: homeobox transcription factor; candidate region associated with feather pecking [51]
chr4:85.64–85.72	CD8A*: role in immunity; regulation of T cells and metabolism [57]
	RMND5A: involved in pyruvate metabolism [58]
chr4:90.16–90.22	CYP26B1: role in metabolism of retinoic acid [59]; involved in controlling the timing of meiosis [60]
chr6:12.88–12.94	KIF20B*: kinesin family member; role in cytokinesis; candidate gene associated with carcass yield in ducks [61]
chr9:24.12–24.16	Gene void: closest gene is ENSGALG00000054892 (lncRNA)
chr10:18.02–18.06	CHSY1: role in biosynthesis of chondroitin sulfate and bone development [62]; candidate gene associated with egg number [63]
	SELENOS: role in degradation of misfolded proteins; endoplasmic reticulum stress regulator; correlated with apoptosis susceptibility [64]
	SNRPA1: role in mRNA splicing; hub gene correlated with phagocytosis [65]; candidate region associated with egg number [63]
chr28:0.14–0.18	Gene void: closest gene is GYG1, a glycogenin member with a role in glycogen metabolism [66]

One of the strongest selective sweeps was detected on chromosome 6 (12.88–12.94 Mb), a region containing two novel genes (*ENSGALG00000050687*, *ENSGALG00000047037*) with homology to kinesin family member 20B (*KIF20B*). As *KIF20B* is involved in cell division and is located near a carcass yield QTL in ducks [61], this signal likely points to selection for growth efficiency. Further strong selective sweeps were detected on chromosomes 28 (0.14–0.16 Mb) and 9 (24.12–24.16 Mb), both of which are located in intergenic regions. The closest gene found in proximity of the candidate region on chromosome 28 is *ENSGALG00000054892*, which is a long non-coding RNA (lncRNA) and as such is potentially involved in regulatory processes [67]. On the other hand, we found *ENSGALG00000035535* (*GYG1*) in closest proximity to the region on chromosome 9. *GYG1* codes for a family member of glycogenin, a protein that is crucial for glycogen biosynthesis in muscle tissues [66] and hence might play an important role for growth and meat quality in chickens.

Consistent with selection for growth and muscle development, we identified several other candidate genes. The gene *DAG1*, a known hub for muscle development [52], and *ASZ1*, located near QTLs for carcass and breast weight [47], were both under selection. We also detected a signal encompassing *ELK3*, a gene previously linked to improved feed conversion ratio in an indigenous Chinese chicken breed [48].

Reflecting the Ixworth breed’s dual-purpose role, we also found strong signatures of selection related to reproduction and skeletal health. A candidate region on chromosome 1 contained *INTS13*, known to be associated with egg weight [54]. On chromosome 10, a sweep overlapped with a known QTL for egg number [63]. Within these regions, we identified genes crucial for skeletal integrity, such as *CHSY1*, which is involved in chondroitin sulfate biosynthesis essential for joint health [68].

Finally, several candidate regions pointed to selection on immunity, metabolism, and behavior. These included *CDK17*, which is upregulated during avian leukosis virus infection [49], and *SNRPA1*, a hub gene regulating the immune process of phagocytosis [65]. Another candidate gene, *SELENOS*, is known to regulate endoplasmic reticulum stress in response to selenium deficiency [64]. Notably, two sweeps on chromosomes 1 and 4 overlapped with QTLs for feather pecking [51], highlighting selection on an important behavioral trait in poultry. The most prominent selective sweep consistently identified in all analyses was found on chromosome 4 around approximately 90.16 Mb, a region that is close to multiple QTLs associated with cooking qualities [69, 70]. In close proximity are *CYP26B1*, a gene involved in retinoic acid metabolism and the timing of meiosis [60], and *EXOC6B*, an exocytosis regulator, associated with egg yolk properties [71].

#### Intense selection for growth and metabolic efficiency in broilers

To map the distinct selection pressures shaping broilers and the dual-purpose Ixworth chicken, we performed cross-population comparisons between Ixworth chicken and two commercial broiler populations. The analysis identified 25 (line A) and 11 (line B) genomic regions under positive selection using the top 0.1 % windows based on $|XP-EHH|_w> 3.32$ and $|XP-EHH|_w> 3.46$, respectively (Supplementary Figures S5 and S6). While the majority of strong signals indicated intense selection for alleles in the broiler lines, we also uncovered key regions where the Ixworth chicken population harbors the selected allele, revealing the nature of their divergent evolution (Supplementary Table S2 and Supplementary Files S4 and S5). A list of key candidate genes related to production, immunity, and behavior is provided in Table 5.

**Table 5:** Candidate regions and genes of interest under positive selection between Ixworth chickens and two commercial broilers according to$|XP-EHH|_w$. Annotations for gene symbols marked by asterisks (“*”) were annotated by BLAST

Comparison	Location (Mb)	Genes and functions
Ixworth vs. broiler line A	chr1:54.32–54.36	ALDH1L2*: role in lipid metabolism; produces NADPH in mitochondria [72]
		SLC41A2: role in magnesium transport; upregulated in uterine cells during eggshell calcification [73]
	chr2:24.66–24.72	C1GALT1: potential role in regulating gut health [74]
		COL28A1*: downregulated when comparing commercial broilers and native chickens [75]; superfamily members associated with abdominal fat (COL12A1) and meat color (COL1A2) [76]
		ASNS: role in biosynthesis of aspartate; potentially regulates growth and muscle development [77]
	chr2:24.84–24.88 & chr2:24.92–25.08	ICA1/UMAD1/GLCCI1: mostly uncharacterized; candidate genes associated with eggshell color [78] and located within ROH island [33]
		NXPH1: role in nervous system and sperm motility [79]
	chr3:3.94–4.00	SLC24A3: role in calcium homeostasis; indirectly related to feed conversion ratio [80]
	chr3:29.50–29.56	DNAH8: role in sperm motility and fertility; downregulated in low sperm motility roosters [81]; previously identified signature of selection between broilers and layers [82]
	chr4:6.14–6.30	Gene void: two lncRNAs; candidate region associated with muscle pH [83] and feather pecking [51]
	chr4:90.24–90.28	Gene void: one lncRNA; candidate region associated with cooking loss [69] and fatty acid atherogenic index [70]
	chr13:0.68–0.74 & chr13:0.82–0.88	PCDHB4*/PCDHB15/PCDHB16*: potential role in development of nervous, musculoskeletal and cardiovascular systems [84]
Ixworth vs. broiler line B	chr1:54.10–54.22	APPL2: multi-functional adapter protein; roles in processes such as cell proliferation, cell metabolism and immune response; candidate region associated with testicular traits [85, 86]
	chr3:27.88–27.92	SLC4A1AP*: role in molecule transport; upregulated when comparing hatched chickens and embryos [87]
		SUPT7L: role in chromatin modification; hub gene for downregulated genes in response to avian influenza [88]
	chr3:28.12–28.64	LRFN2: role in nervous system; upregulated when comparing high and low egg laying ducks [89]; candidate region associated with body weight [90]
	chr10:19.34–19.38	IQCH*: potential role in fertility via sperm motility [91]
		C15orf61: uncharacterized; candidate region associated with yolk water content [92]
	chr18:0.48–0.52	MYH1B: role in skeletal muscle development and adipogenesis [93]
	chr18:0.66–0.70	SCO1*: role in cellular copper homeostasis and mitochondrial respiratory chain [94]
		ADPRM: potential role in immune cell signaling and cellular metabolism [95]
Shared	chr4:90.10–90.18 & chr4:90.10–90.20	CYP26B1: role in metabolism of retinoic acid; involved in controlling the timing of meiosis [60]
		EXOC6B*: role in cell growth, polarity, and migration; associated with thermogelled egg yolks [71]
	chr26:0.00–0.04	LRMPL: lymphoid-restricted membrane protein-like; potential role in immunity [96]
		MICAL1: potential role in growth; downregulated when comparing fast and slow-growing broilers [97]

The most prominent pattern was selection favoring broiler alleles related to rapid growth. A strong, shared selective sweep in both broiler lines relative to the Ixworth chicken was found on chromosome 26 (0.00–0.04 Mb), a region containing *MICAL1*, a gene whose expression is linked to growth rate differences [97]. Additionally, line-specific sweeps targeting growth and development were evident. In line A, a sweep on chromosome 13 contained genes from the protocadherin beta family (*PCDHB*), critical for nervous system and musculoskeletal development [84]. In line B, a strong signal on chromosome 3 (27.88–27.92 and 28.12–28.64 Mb) intersected *LRFN2* and a QTL which was shown to be associated with body weight [90].

Both broiler lines showed convergent selection for enhanced metabolic efficiency, though via different genetic pathways. In line A, selection favored alleles in *ALDH1L2* (lipid metabolism) [72] and *ASNS* (amino acid biosynthesis) [77]. In parallel, line B showed strong selection for alleles in *SCO1* (copper homeostasis) [94]. Further broiler-specific signals were linked to muscle development (*MYH1B* in line B) [93] and meat quality, including QTLs for muscle pH and cooking loss (line A) [69, 70, 83].

In contrast to the dominant broiler trends, our analysis revealed a region on chromosome 3 (3.94–4.00 Mb) where selection strongly favored the Ixworth chicken’s allele over broiler line A. This Ixworth-specific sweep contains *SLC24A3*, a gene involved in intracellular calcium homeostasis with a potential link to feed conversion ratio [80].

Finally, we identified several sweeps in broilers encompassing genes implicated in reproduction and behavior, likely representing responses related to intense growth selection. These included broiler-favored alleles in genes related to sperm motility (*DNAH8*, *NXPH1*, *IQCH*) [79, 81, 91], eggshell quality (*SLC41A2*) [73], and testis development (*APPL2*) [85, 86]. Notably, a sweep in line A also overlapped a QTL on chromosome 4 associated with feather pecking [51], highlighting the complex genetic trade-offs between extreme growth and other fitness-related traits.

#### Intense selection for reproductive efficiency in layers

To investigate selection pressures related to egg production, we compared the Ixworth chicken to two commercial layer lines (Table 6). The analysis identified 16 (brown) and 23 (white) genomic regions under positive selection using the top 0.1 % windows based on $|XP-EHH|_w> 3.10$ and $|XP-EHH|_w> 2.92$, respectively (Supplementary Figures S5 and S6). In contrast to the broiler comparisons, which were dominated by strong sweeps favoring the commercial lines, this analysis revealed a similar trend for the brown layer comparison but more balanced sweeps in the white layer comparison (Supplementary Table S2 and Supplementary Files S6 and S7).

**Table 6:** Candidate regions and genes of interest under positive selection between Ixworth chickens and two commercial layers according to $|XP-EHH|_w$. Annotations for gene symbols marked by asterisks (“*”) were annotated by BLAST

Comparison	Location (Mb)	Genes and functions
Ixworth vs. brown layer	chr1:171.16–171.22	Gene void: five lncRNAs; candidate region associated with meat-related traits [98]; closest genes are DLEU7* and RNASEH2B; RNASEH2B is associated with growth-related traits [99]
	chr1:171.84–171.90	FOXO1: role in immunity; regulator in ovarian follicles for apoptosis, stress response, hormonal signaling [100]; negative regulator of skeleton muscle mass in mice [101]
		SLC25A15: role in urea cycle; under selection related to body size and egg production [102]; involved in ammonium detoxification affecting laying performance [103]; candidate region associated with meat-related traits [98]
	chr4:90.10–90.18	CYP26B1: role in metabolism of retinoic acid; involved in controlling the timing of meiosis [60]
		EXOC6B*: role in cell growth, polarity, and migration; associated with thermogelled egg yolks [71]
	chr5:3.06–3.12	SLC17A6: role in L-glutamate transport into synaptic vesicles; potentially related to feather picking [104]; potentially selected in heat adaptation [105]
	ch5:4.16–4.22	METTL15P1: role in rRNA modification (pseudogene); potentially selected for adaptation to harsh environments [39]
	chr5:5.22–5.26	DNAJC24: role in post translational modification; associated with yolk weight in ducks [106]
		IMMP1L: role in mitochondrial protein maturation; associated with bone quality traits during egg-laying periods in ducks [107]
	chr5:7.36–7.48	SYT9: role as a calcium-dependent neurotransmitter; candidate region associated with intramuscular fat percentage [108]
	chr5:7.74–7.78	FAR1: role in lipid metabolism; regulates follicular development in goose [109]
	chr13: 0.68–0.74	PCDHB4*/PCDHB15/PCDHB16*: potential role in development of nervous, musculoskeletal and cardiovascular systems [84]
	chr33:7.40–7.44	PCBP2: involved in many functions, including immunity; downregulated during eggshell formation [110]
		MAP3K12: role in signal transductions; downregulated during Newcastle Disease infection [111]
Ixworth vs. white layer	chr1:147.82–147.88	SOX21: involved in regulation of various developmental processes, including inner ear development [112]
		GPR180*: potential role in vascular remodeling and lipid metabolism; associated with body weight in goose [113]
		TGDS*: role in various metabolic processes; candidate region associated with neck weight [98]
	chr3:4.08–4.14	SLC24A3: role in calcium homeostasis; indirectly related to feed conversion ratio [80]
		RIN2*: role in regulation of endocytic membrane trafficking; potentially related to fat deposition [114]
	chr3:9.78–9.82	EHBP1*: involved in endocytic trafficking; candidate region under selection for abdominal fat content [115, 116]
	chr5:34.40–34.44	SCFD1: role in autophagy; associated with intramuscular fat [117]
	chr5:37.92–37.98	COQ6*: role in biosynthesis of coenzyome Q10, preventing oxidative stress; downregulated when using thermal manipulation during embryogenesis [118]
		ALDH6A1*: role in catabolic pathways; hub gene associated with abdominal fat [119]
		ENTPD5: potentially involved in catabolism of extracellular nucleotides; candidate region associated with antibody titer to KLH antigen [120]
	chr5:38.08–38.12	VRTN: involved in embryonic development; associated with vertebral number in pigs [121]
		SYNDIG1L*: role in the nervous system; associated with egg weight in ducks [106]
	chr7:36.18–36.22	GALNT5*: involved in metabolism; candidate region in proximity associated with body weight and age at first egg [122, 123]
	chr9:22.58–22.70	RSRC1: role in alternative mRNA splicing; associated with bone quality traits [124]
		RARRES1: role as retinoid acid receptor responder; highly expressed in uterus related to cuticle deposition [125]
	chr12:19.02–19.06	ARL8B: involved in GPT binding; role in innate immunity; potentially associated with egg number [63]
		EDEM1: role in calcium ion binding and mannosyl-oligosaccharide 1,2-$\alpha$-mannosidase activity; potentially associated with egg number [63]
	chr17:9.80–9.92	RPL35: ribosomal protein; potential role as immunity biomarker [126]
		SCAI: role in cell migration regulation; candidate region associated with eicosenoic acid content and feather pecking [51, 70]
	chr19:3.38–3.42	ZZEF1: role in chromatin organization; involved in calcium ion binding; candidate region associated with heterophil/lymphocyte ratio [127]
	chr33:5.40–5.44 & chr33:5.46–5.52	ITGA7*: involved in cell-cell and cell-matrix interactions; related to muscular disorders; candidate gene for egg yolk deposition [128]
		CD63: role in regulation of cellular processes and innate immunity [129]
Shared	chr5:6.00–6.04 & chr5:5.92–6.04	DYNC1LI2-like*: involved in mitosis [130]; a potential ortholog is associated with growth [131]
	chr5: 6.52–6.56	Gene void: six lncRNAs

A prominent pattern was strong selection on genes crucial for reproductive efficiency in both layers. Selection favored layer-specific alleles in genes directly implicated in egg production, such as *FOXO1* and *SLC25A15* (follicular development and laying performance) [100, 102, 103] in the brown line, and *ITGA7* (yolk deposition) [128] in the white line. The brown line, in particular, showed target pressure on multiple reproductive traits, with additional sweeps on *FAR1* (follicular development) [109], *PCBP2* (eggshell formation) [110], and *DNAJC24* as well as *IMMP1L* (egg and bone quality in ducks) [106, 107].

Strikingly, when compared against the highly specialized white line, selection favored the Ixworth chicken’s alleles for a suite of critical reproduction genes. These included *RARRES1*, involved in uterine function and cuticle deposition [125], as well as *ARL8B* and *EDEM1*, both of which are associated with egg number [63]. This unexpected finding suggests that while commercial lines have been optimized for a specific reproductive strategy, the Ixworth chicken may have retained alternative advantageous genetic variants for maintaining robust, long-term laying performance.

Complementing the focus on reproduction, both layer lines showed selection for metabolic pathways supporting high output while regulating body composition. The white line exhibited sweeps on genes that affect fat deposition, such as *EHBP1* [115, 116], and a region near *GALNT5* associated with body weight and age at first egg [122, 123]. This line also showed selection on *SOX21*, *GPR180*, and *TGDS*, which are genes involved in metabolism of lipids and proteins and associated with inner ear development [112] and neck weight in chicken [98] as well as body weight in goose [113]. The brown line, however, showed a more complex pattern, with selection on a major growth and meat QTL on chromosome 1 (171.16–171.90.16.90 Mb) [98, 99]. This region contains genes like *RNASEH2B* (growth) [99], *FOXO1* (immunity and reproduction) [101], and *SLC25A15* (body size and egg production) [102, 103], suggesting that the brown layer’s selection history may have involved a different trade-off between body size and egg production compared to the more specialized white line. Furthermore, in the brown line, selection targeted metabolic pathways through two proximal regions on chromosome 5. The first region (7.36–7.48 Mb) contains the neurotransmitter *SYT9* and is associated with intramuscular fat percentage [108], and the second region (7.74–7.78 Mb) contains *FAR1*, a gene that also influences lipid metabolism [109].

Despite their differences, evidence for a shared layer-specific selection strategy was found on chromosome 5, where both lines exhibited strong sweeps relative to the Ixworth chicken. One shared region (5.92–6.04 Mb) contains a *DYNC1LI2*-like protein, a gene involved in mitosis [130]. A structurally similar *DYNC1LI2* protein was previously identified close to a QTL on chromosome 11 associated with growth [131]. A second nearby shared region contained several lncRNAs, suggesting that selection in layers has also targeted regulatory elements.

#### Genomic regions under divergent selection since domestication

To identify genomic regions that have diverged since domestication, we compared the Ixworth chicken to a red junglefowl reference population. We identified 21 significant regions (Supplementary Table S2 and Supplementary File S8) of divergence (top 0.1 % of windows) based on $|XP-EHH|_w> 3.71$ (Supplementary Figures S5 and S6). It is important to note that the red junglefowl used for sequencing originates from a captive, isolated population that may include introgression from domesticated or feral stocks and therefore might not be fully representative of wild birds [1]. Thus, this comparison highlights strong signals of divergence on genes that have occurred during the Ixworth chicken’s specific formation as a dual-purpose breed, though some signals may stem from the partly domesticated nature of the red junglefowl population (Table 7). However, the vast majority of the strongest signals indicated long, ancestral haplotypes retained in the red junglefowl population, while the Ixworth chickens carried alternative, derived alleles, in line with the observation that red junglefowl carry a relatively higher number of ROH segments above 1 Mb (Table 3). This pattern, most pronounced on gene-dense microchromosomes (Table 7), likely reflects demographic history combined with natural selection on functional regions, particularly genes related to survival and immunity, rather than purifying selection typically observed in domesticated chicken [1].

**Table 7:** Candidate regions and genes of interest under positive selection between Ixworth chickens and red junglefowl according to $|XP-EHH|_w$. Annotations for gene symbols marked by asterisks (“*”) were annotated by BLAST

Location (Mb)	Genes and functions
chr2:131.12–131.16	PYGM*: glycogen phosphorylase, muscle form-like; annotations absent in birds; enzyme involved in glycogenolysis in skeletal muscle [132]
chr4:90.10–90.24	CYP26B1: role in metabolism of retinoic acid; involved in controlling the timing of meiosis [60]
	EXOC6B*: role in cell growth, polarity, and migration; associated with thermogelled egg yolks [71]
chr4:90.26–90.36	Gene void: four lncRNAs; candidate region associated with cooking loss [69] and fatty acid atherogenic index [70]
chr4:90.46–90.50	Gene void: three lncRNAs; candidate region associated with cooking loss [69]
chr10:0.60–0.66 & chr10:0.86–0.92	ANXA4*: involved in membrane repair; potential role in regulation of exocytosis [133]
chr13:0.74–0.78 & chr13:0.82–0.88 & chr13:0.90–0.94 & chr13:1.08–1.14	PCDHB4*/PCDHB15*: potential role in development of nervous, musculoskeletal and cardiovascular systems [84]
chr13:2.42–2.48	PCDHGA5*/PCDHGA10*: role in development of nervous system; involved in neuronal survival and synapse formation [134]
chr15:0.00–0.04	FIS1*: involved in mitochondrial fission; potential role in energy metabolism; upregulated in pectoralis muscle when comparing migratory and non-migratory birds [135]
	DGAT2*: role in metabolism; involved in fat storage; associated with carcass and meat quality in domestic pigeons [136]
chr15:0.10–0.14	MOGAT2*: role in metabolism; involved in dietary fat digestion and absorption [137]
	FGF11*: potential role in nervous system; involved in many biological processes such as embryonic development and cell growth [138]
	NLGN2*: potential role in nervous system; involved in synaptic signal transmission; annotations absent in chicken [139]
chr23:0.02–0.12	ANXA4*: see above
	BLOC1S1*: role in endosome–lysosome and vesicle trafficking; involved in modulation of metabolic pathways [140]
chr23:5.96–6.02	HLA-DRA*: part of the major histocompatibility complex; role in (adaptive) immune system [141]
chr24:6.34–6.50	CHIR2D*: uncharacterized; similar to immunoglobulin-like receptor, typically found on chromosome 31; potential role in immune response [142]
	CLPTM1*: role in the nervous system [143]
	MVP*: role in immune system and multidrug resistance; potentially involved in viral defense [144]

The most powerful signals of divergence were found on microchromosomes 23 and 24, where the red junglefowl harbored extended homozygous haplotypes. These regions contain dense clusters of genes potentially relevant for survival in a natural environment, including functions related to immunocompetence and stress sensitivity, that might have undergone change as a result of relaxed or directed selective pressures of managed environments [145]. A sweep on chromosome 24 (6.34–6.50 Mb) favored red junglefowl alleles at a locus including *MVP* (multidrug resistance, immune system) [144], *CLPTM1* (nervous system) [143], and *CHIR2D* (immune system) [142], a putative immunoglobulin-like receptor. Similarly, a sweep on chromosome 23 (5.96–6.02 Mb) favored red junglefowl alleles intersecting *HLA-DRA*, important for the adaptive immune system [141], and *ANXA4*, involved in membrane repair and implicated in exocytosis [133].

In line with the dominant pattern of strong selection on the ancestral alleles of red junglefowl, we identified several sweeps related to a variety of metabolic processes. On chromosome 15 (0.00–0.04 Mb), sweeps on *DGAT2* and *MOGAT2* point to regulation of dietary fat digestion and absorption as well as storage and were previously linked to carcass quality in pigeons [136, 137]. Furthermore, putative protocadherin gene clusters on chromosome 13 (0.74–1.12 Mb and 2.42–2.48 Mb), implicated in neurodevelopment [84, 134], also showed selection in the red junglefowl population. Notably, this region was also detected across comparisons of Ixworth and brown layers as well as broiler line A, suggesting its potential as a target for future investigations into behavioral traits. Various other sweeps were also detected involving genes related to regulation of the nervous system, such as *FGF11* [138] or *NLGN2* [139], and hence could play a role in the behavioral differences of wild and domesticated chicken.

Finally, reaffirming its central role in defining the Ixworth chicken’s unique genetic identity, the region on chromosome 4 containing *EXOC6B* and *CYP26B1* was once again a strong target of selection in the Ixworth chicken relative to red junglefowl. Its consistent differentiation from the wild ancestor, as well as from specialized commercial lines, except for the white layer, solidifies its status as a core signature region defining the Ixworth chicken’s genetic trajectory as a local dual-purpose breed.

## Discussion

This study presents a comprehensive genomic analysis of the Ixworth chicken, providing critical insights into its genetic architecture compared to specialized commercial lines and its wild progenitor. Our findings reveal that the Ixworth chicken possesses a unique genetic profile characterized by moderate to high genetic diversity, a distinct population structure, and a balanced selection history consistent with its local dual-purpose background. These findings are particularly interesting due to the limited population size and endangered status of the breed.

### Genetic diversity and population structure of a dual-purpose breed

The Ixworth chicken occupies an intermediate position in the spectrum of chicken genetic diversity. With 11.59 million SNPs and over 510 thousand private alleles, its genetic variability is substantially higher than that of specialized layer lines but lower than that of red junglefowl (Table 2). This is enforced by metrics such as moderate nucleotide diversity and low inbreeding coefficients. Furthermore, its observed heterozygosity was slightly higher than expected, indicating a minor excess of heterozygotes, a pattern consistent with random mating and less intensive breeding.

The population structure of Ixworth chicken is similarly nuanced. While the PCA initially places Ixworth chickens in proximity to brown layers in the first two PCs (Fig. 1a), their relationship is more complex, as revealed in higher PCs (Supplementary Figure S1). This complexity is underscored by a lower genetic differentiation between Ixworth chicken and both broiler lines and red junglefowl compared to its differentiation from layers. Admixture analysis confirms this, identifying Ixworth chicken as a genetically distinct population without evidence of recent admixture at higher k-values (Fig. 1c). Exploring lower k-values reveals a shifting ancestral picture: at $k = 5$, both broiler lines share the same cluster, at $k \le 4$, Ixworth chickens share a closer ancestral component with broilers, while at $k = 3$ a potential relationship with brown layers emerges, before layer populations merge at $k = 2$ with some admixture with broilers. This suggests a complex ancestral history of the Ixworth chicken rather than a simple common ancestry. The emergence of potential subpopulation structures at higher k-values, while not conclusive, could be expected, given that genetic variation within livestock populations is often high and complex due to their unique domestication and breeding histories [146].

The decay of LD provides a clear historical context for these structural patterns, a nuanced lens into the demographic and selection history of each population. In Ixworth chicken, LD decays rapidly, following the patterns observed in the red junglefowl and the commercial broilers. This rapid decay is characteristic of local breeds with larger effective populations or a less intensive selection history [40, 147]. Our findings align with observations from other indigenous poultry populations. For example, studies on African village chickens have documented similarly low mean LD and high levels of genetic polymorphisms compared to more managed stocks [148]. The higher level of polymorphisms in these local flocks, reflected in fewer monomorphic markers, is a direct parallel to what we observe when comparing the diverse Ixworth chicken genome to the more homogeneous layer lines. For Ixworth chicken, this rapid LD decay signifies high haplotype diversity and shorter haplotype blocks, directly in line with the conclusions drawn from our heterozygosity analysis. In contrast, the layer lines exhibit extensive LD and a much slower decay, a well-documented genomic result of intensive selection [149]. Such high background LD is a clear signature of historical bottlenecks, non-random mating, and genetic drift, which collectively reduce haplotype diversity. These phenomena are the expected consequences of breeding programs that rely on small $N_e$ and strong directional, recent artificial selection for a narrow set of traits [148, 149]. Therefore, the LD profile confirms that the Ixworth chicken has maintained a more diverse genetic base, having avoided the extreme selection pressures and constrictions that have profoundly reshaped the genomes of many modern commercial lines. However, despite the observed patterns of moderate to high diversity, low inbreeding, and rapid LD decay, the Ixworth chicken is expected to exhibit low $N_e$, largely due to its limited availability, small population sizes, and endangered status [11], factors that may restrict the potential for sustained genetic diversity in the future. To assess the long-term viability of the breed, accurate estimates of contemporary $N_e$ may become necessary.

### Historical context and divergent paths of specialization

The distinct genetic profile of the Ixworth chicken becomes clearer when placed in its historical context alongside other British breeds and its commercial counterparts. Previous phylogenetic work identified the Light Sussex, a classic English dual-purpose breed with similar production characteristics, as the closest relative to the Ixworth breed [12]. Other breeds like Croad Langshan, Buff Orpington, and Indian Game show a more distant ancestry, highlighting a shared history of dual-purpose, meat, or exhibition roles among older British poultry [12].

Of particular relevance to our study is the Rhode Island Red, which we referred to as the brown layer in this study. Although it clusters near Ixworth chickens in the PCA plot (Fig. 1a), its high genetic differentiation and extensive LD reflect a different evolutionary trajectory. Originally a dual-purpose breed itself, the Rhode Island Red was intensively re-developed into the foundation for commercial brown layers starting in the 1940s [12, 150]. In contrast, the White Leghorn, which we referred to as white layer, showed the highest differentiation among all populations and the highest levels of inbreeding. It was historically influenced by a primary breeding focus on egg-laying performance [150]. Although white and brown layer lines are highly differentiated from each other, this divergence primarily reflects specialization rather than elevated within-line diversity. Intensive selection has led to strong line-specific haplotype fixation in both populations, as demonstrated by reduced genetic variation, slow LD decay, and distinct selection signatures. Our findings are consistent with previous research reporting low correlation between LD structures of white and brown layers [151]. This showcases how a breed’s genetic potential, despite having different backgrounds, can be channeled towards a single purpose, such as laying performance, often at the direct expense of genetic diversity, which the Ixworth chicken has retained.

While genetic divergence from layers was expected, the commercial broiler lines surprisingly exhibited high genetic diversity, with rapid LD decay and haplotype patterns similar to the non-commercial Ixworth chicken and red junglefowl populations. This contradicts the expectation of low diversity in highly specialized commercial chickens [152]. The explanation most likely lies in the different origins of the sampled populations. The broiler lines were historical sire lines established in the 1970s and 1980s from large founding populations [1]. In contrast, the layer pure lines were sampled from more modern breeding programs, which use a small number of founders and selective breeding with limited $N_e$ [1, 33]. It is therefore plausible that the broiler lines originate from a broader genetic base and do not represent the genomic architecture of today’s highly-specialized commercial stock. Although both broiler and layer breeding involve strong selection, these historical differences in population management and era may explain the observed variations in genetic diversity.

### Genomic signatures of specialization and domestication

To understand the functional consequences of these distinct evolutionary trajectories, we investigated signatures of selection. As expected, the commercial lines displayed strong selective sweeps in genomic regions containing genes crucial for their specialized functions. These provide us with information on two intertwined aspects. First, we identify the evolutionary trajectories that have been shaped by artificial selection of broilers and layers in contrast to the Ixworth chicken. Second, these regions can help guide future selection schemes for the Ixworth chicken, focusing on both traits.

- Broilers: Both broiler lines displayed strong selection signals in genomic regions associated with growth and metabolism. These were particularly pronounced in broiler line A, which showed selective sweeps on chromosomes 2 and 4 on regions that harbor meat-production related genes such as *COL28A1* [76] and *ASNS* [77] (Table 5), as well as a locus, previously associated with muscle pH and feather pecking behavior [51, 83], which is also containing lncRNAs. Similarly, broiler line B has experienced strong selection pressure on chromosome 3, in a region containing the *LRFN2* gene, which is known to be associated with body weight [90]. A shared signal of both lines was found at the start of chromosome 26, encompassing the *MICAL1* gene. Notably, *MICAL1* was previously found to be downregulated in a similar comparison of fast-growing broilers and slow-growing broilers [97], suggesting its role in modulating growth rates.
- Layers: Consistent with a previous study by Sallam et al. [151], we observed high population separation between brown and white layer lines, given strong clustering in the PCA plot and strong genetic differentiation. This divergence in their genomic landscapes provides a framework to contrast distinct selection histories, corresponding to differing production goals and breeding strategies. The brown layer’s (Rhode Island Red) history as a dual-purpose bird is reflected in its genome in the form of strong selective sweeps. A key region on chromosome 5, encompassing genes like *DNAJC24*, *IMMP1L*, *SYT9*, and *FAR1*, links selection to both reproductive fitness (follicular development, yolk weight) [106, 109] and physical robustness (bone quality, fat percentage, behavior) [104, 107, 108]. A second major sweep on chromosome 1, targeting *FOXO1* and *SLC25A15*, further reinforces its dual-purpose history by implicating genes that regulate immunity, body size, and muscle mass alongside ovarian function [100–102]. In contrast, the white layer genome reveals a modern, highly specialized breeding strategy. Instead of large, concentrated sweeps, we observed more diffuse selection signals across the genome, yet they consistently fall in regions associated with egg production. Selected genes such as *SLC24A3*, *ARL8B*, *EDEM1*, *ITGA7*, *EHBP1*, and *GALNT5* are associated with key efficiency traits, including feed conversion, abdominal fat content, and an early onset of laying [63, 80, 122, 123, 128]. This genomic architecture strongly suggests an intensive selection pressure that has fine-tuned the White Leghorn into a more efficient egg producer, while the Rhode Island Red also prioritized fitness traits.
- Red junglefowl: The red junglefowl provides a baseline of adaptation to a natural environment, contrasting with the domesticated Ixworth chicken. We detected various selection signals on genes, potentially crucial for survival in the wild or as an adaptation to managed farming. For instance, we identified strong pressures on immunity and metabolism, targeting genes like *HLA-DRA* [141], *MVP* [144], *DGAT2* [136], and *MOGAT2* [137], many of which reside on microchromosomes. This highlights the need for a robust immune system and efficient energy regulation in a challenging environment. At the same time, selection has shaped genes associated with the nervous system (*PCDHGA* [134], *NLGN2* [139], *FGF11* [138]), likely adaptations for behaviors critical for flock integration and survival. These signatures of wild-type fitness were less present in the Ixworth chicken, suggesting a domestication trade-off. As artificial selection prioritized production traits under the buffered conditions of managed farming, alleles essential for wild survival and behavior were likely lost or selected against.

### Balanced selection in the Ixworth chicken

The genomic architecture of the Ixworth chicken assembles into a balanced image of selection on both egg and meat traits, telling a story about its history as a classic dual-purpose breed. Rather than the uniform pattern of a specialist, we see a complex design balancing productivity, resilience, and unique quality traits. Dual-purpose genetics are particularly intricate because growth and reproductive traits have historically shown negative genetic correlations under strong directional selection in specialized lines, leading to trade-offs when single traits are prioritized [153, 154]. In commercial breeding practice, however, these trade-offs are mitigated through multi-line hybrid breeding schemes in which growth performance and reproductive efficiency are jointly optimized across parental lines rather than within a single population. Dual-purpose breeds such as the Ixworth represent a distinct breeding paradigm, in which these traits are selected within the same genetic background, without reliance on hybridization between specialized lines. Consistent with this, a recent study did not observe a negative correlation between growth and reproductive traits in Ixworth chickens [11], indicating that antagonistic relationships described in specialized lines may not directly translate to balanced dual-purpose populations.

Furthermore, dual-purpose chickens such as the Ixworth are frequently associated with favorable welfare and health traits, including lower mortality and a reduced incidence of behavior-related disorders, reflecting their moderate growth and balanced selection regime [11]. At the same time, production-associated disorders, including growth-related myopathies, remain relevant across poultry systems and are reported more frequently in fast-growing broiler lines, with prevalence influenced by both genetic background and management conditions [155]. Although the underlying mechanisms linking growth rate and myopathies are not yet fully understood [155], slower-growing genotypes typical of dual-purpose chickens may contribute to reduced prevalence rather than complete avoidance. Importantly, welfare- and health-related disorders are not absent in dual-purpose or local populations, and conditions such as keel bone fractures and other diseases have also been documented, albeit generally at lower prevalence [156]. As these traits are inherently multi-factorial and beyond the scope of the present study, dual-purpose populations may nonetheless represent a valuable genetic resource for breeding strategies aiming to balance performance, welfare, and health objectives under changing production demands [157].

In our investigation, we identified positive selection on key production traits in the Ixworth chicken population, distributed across multiple chromosomes. For meat productivity, we identified sweeps on chromosomes 1, 6, and 28, implicating genes known to influence efficient growth and carcass characteristics, such as *ASZ1*, *ELK3* (feed conversion, growth) [47, 48], *KIF20B* (carcass yield) [61], and *GYG1* (glycogen metabolism). Set against these are counterbalancing loci for egg-laying performance on chromosomes 1, 3, and 10, near genes like *INTS13* and *CHSY1* (egg weight and number) [54, 63], and *NKAIN2* (age at first egg) [56]. The selection for robust immunity (*CDK17*, *CD8A*) [49, 57] and behavior [51] on chromosomes 1 and 4 is complementing the production-focused design.

However, the centerpiece of its genomic architecture is a unique selective sweep on chromosome 4 (approx. 90.10–90.30 Mb), a signature that sets the Ixworth chicken apart from the other populations. This region is a functional hotspot, containing candidate genes including *EXOC6B* (*ENSGALG00000016098*) for cell growth and, most notably, *CYP26B1* (*ENSGALG00000016102*), a critical modulator of retinoic acid metabolism vital for embryonic development. In addition, the region is densely populated with numerous lncRNAs and microRNAs, suggesting it functions not just as a collection of individual genes, but also as a sophisticated regulatory hub. Intriguingly, QTLs for both cooking loss [69] and the fatty acid atherogenic index [70] are located within the same locus on chromosome 4. Pampouille et al. [69] discussed potential pleiotropic effects on this cooking loss QTL, highlighting the importance of the nearby dysferlin gene and its interaction partners, whose mutations are implicated in muscular dystrophy. Similarly, Fan et al. [70] identified QTLs on chromosome 4 associated with fatty acid composition in the breast muscle of Gushi chicken, a local Chinese breed known for its high-quality meat, possibly comparable to that of the Ixworth chicken. Hence, the force of selection on this locus might imply that Ixworth chicken breeders targeted a fundamental developmental or growth pathway. This alignment provides a compelling explanation for another key piece of the Ixworth breed’s identity and raises questions about the breeders’ selection intent.

In the early 20th century, before the focused shift to specialized lines, dual-purpose breeds were valued for their all-around utility. While breeders of this era lacked modern metrics, they could have engaged in empirical selection for superior culinary qualities, choosing birds with juicier meat (lower cooking loss) and richer taste (influenced by fatty acid profiles). Alternatively, the improvement in meat quality may have been an unintentional consequence of selection on this locus for another trait. It is conceivable that the intense selection on this developmental and regulatory hub had pleiotropic effects on meat quality, or vice versa. The genes governing these culinary traits may have simply been swept along with the primary selection target due to tight physical linkage to the related developmental pathway. Nevertheless, this finding sheds light on the breed’s anecdotal reputation for premium table quality, which has been documented for over a century [158]. The qualities of the Ixworth chicken are tangibly written into its genome, partially rooted in this distinct selective sweep, whether by deliberate design or by a fortunate accident of genetic hitchhiking.

### Limitations

While this study provides a high-resolution view of the Ixworth chicken genome, certain methodological limitations should be acknowledged. Given the non-random and sex-biased sampling, with limited cohort information, genomic estimates of $N_e$ were found to be highly sensitive to sampling noise and potentially prone to downward bias. Accordingly, we focused our analysis on direct summaries of genetic differentiation, diversity, inbreeding, and LD structure. These parameters are less assumption-dependent and provide a more robust representation of the observed genomic data in these populations. In addition, while our approach to identifying selective sweeps by aggregating SNP-based statistics (iHS and XP-EHH) into windows is effective for detecting robust signals and a commonly used outlier detection method [40, 159–161], it may oversmooth narrow, recent sweeps or miss signals that fall below our stringent thresholds. Furthermore, functional annotations relied on homology searches (BLAST) against other avian species, as many identified genes were novel in the chicken reference genome (GRCg6a), which carries a risk of misinterpretation.

## Conclusions

In conclusion, this study showcases the Ixworth chicken not merely as a heritage breed, but as a reservoir of genetic diversity largely erased from modern commercial lines. The Ixworth’s genomic composition is characterized by low inbreeding and balanced selection for growth, laying performance, and robustness, which captures the intent to unite productivity and resilience. Notably, this is in line with previous phenotypic data and confirms the dual-purpose potential of the Ixworth breed. Ixworth roosters achieve competitive carcass yields for slow-growing systems while maintaining low mortality and hens show consistent laying performance with high nest acceptance and low incidence of welfare issues. However, it is the distinct selective sweep identified on chromosome 4 (90.10–90.30 Mb), potentially linked to its anecdotal table quality, that might have shaped its unique evolutionary trajectory. The overall genetic profile makes the Ixworth chicken breed a prime candidate for sustainable and ecological farming systems that value animal welfare and independence. The high-resolution genomic data presented here provides a valuable resource for future studies. It enables more precise phylogenetic analyses, imputation for broader genetic studies, and genome-wide association studies focused on validating the novel candidate genes under selection. Ultimately, this genomic information can guide conservation efforts and assist balanced breeding programs aimed at enhancing the productivity of the Ixworth chicken without compromising the valuable genetic diversity it retains.

## Supplementary Information

Additional file 1: Figure S1. Principal component analysis of chicken populations. The first five principal components are shown, explaining 7.94 %, 5.35 %, 4.74 %, 4.54 %, and 3.99 % of the total genotypic variance. Figure S2. Heatmap of chromosome-wise fixation index (weighted $F_{ST}$) estimations between chicken populations. Figure S3. Cross-validation error for ADMIXTURE runs with increasing number of clusters (k). The lowest cross-validation error is reached at $k = 6$. Figure S4. Heatmap of chromosome-wise nucleotide diversity ($\pi$) estimations within chicken populations. Figure S5. The strongest signatures of selection according to $|XP-EHH|_w$ between the Ixworth chicken population and (a) commercial broiler line A, (b) commercial broiler line B, (c) commercial brown layers, (d) commercial white layers, and (e) red junglefowl. Strong selective sweeps are indicated by the red lines according to the top 0.1 %: (a) $|XP-EHH|_w> 3.32$, (b) $|XP-EHH|_w> 3.46$, (c) $|XP-EHH|_w> 3.10$, (d) $|XP-EHH|_w> 2.92$, and (e) $|XP-EHH|_w> 3.71$, respectively. Figure S6. Directional distribution of $XP-EHH_w$ between the Ixworth chicken population and (a) commercial broiler line A, (b) commercial broiler line B, (c) commercial brown layers, (d) commercial white layers, and (e) red junglefowl. A positive direction represents a selective sweep in the Ixworth chicken population (reference) while a negative direction indicates selective sweeps in the other populations. File S1. Relationship between the inbreeding coefficients *F* and $F_{ROH}$. Pearson’s correlation coefficient and linear regression are estimated separately for each population. File S2. Chromosome-wise estimations of LD decay for each chicken population. File S3. Distribution of iHS values within the strongest signatures of selection in the Ixworth chicken. A positive direction represents a selective sweep on the ancestral allele while a negative direction indicates selective sweeps on the derived allele of the Ixworth chicken. File S4. Distribution of XP-EHH values within the strongest signatures of selection in the comparison of Ixworth and broiler line A. File S5. Distribution of XP-EHH values within the strongest signatures of selection in the comparison of Ixworth and broiler line B. File S6. Distribution of XP-EHH values within the strongest signatures of selection in the comparison of Ixworth and brown layer. File S7. Distribution of XP-EHH values within the strongest signatures of selection in the comparison of Ixworth and white layer. File S8. Distribution of XP-EHH values within the strongest signatures of selection in the comparison of Ixworth and red junglefowl. Table S1. Alignment and variant calling statistics. Parameters are calculated as means for each chicken population. Table S2. Genomic location of the strongest signatures of selection (in 40 kb windows) according to the top 0.1 % of the empirical distributions of $|XP-EHH|_w$ and $|iHS|_w$. Directly adjacent windows were merged and intersected with genes. Annotations for gene symbols marked by asterisks (“*”) were annotated by BLAST.

## Data Availability

To promote reproducibility and facilitate examination of our results, the methodology of this study, including reference genome alignment, joint genotyping, population genetics analyses, and further resources are available at https://github.com/Kirschluft/ixworth_popgen. The whole-genome resequencing data used in this study have been deposited in the European Nucleotide Archive (https://www.ebi.ac.uk/ena) under study accession number PRJEB89160.
