# Supplementary material for: Genomic analysis of the Ixworth chicken: insights into a local dual-purpose breed
Source: BMC Genomics. 2026 Mar 11;27:362. doi: 10.1186/s12864-026-12732-9 (PMC13064311; doi:10.1186/s12864-026-12732-9)

Distribution of XP-EHH values within the strongest signatures of selection in the comparison of Ixworth and brown layer

Selective sweep at chr 1:171.16-171.2 Mb with $|XP\text{-}EHH|_w = 3.13$

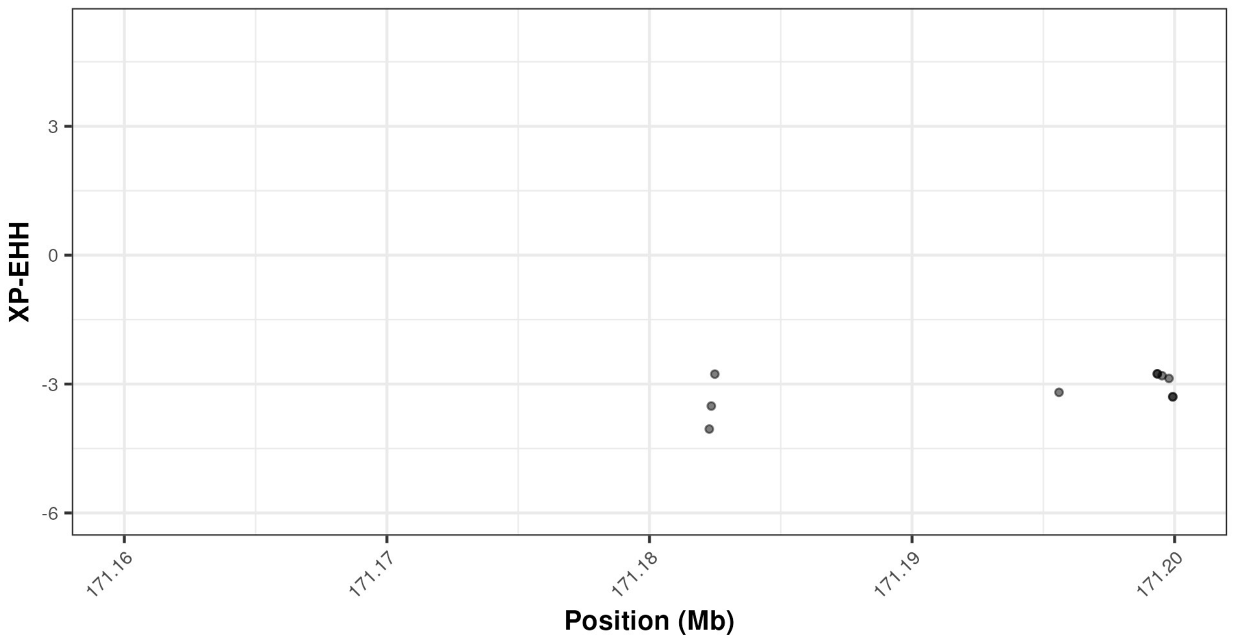

Selective sweep at chr 1:171.18-171.22 Mb with $|XP\text{-}EHH|_w = 3.1$

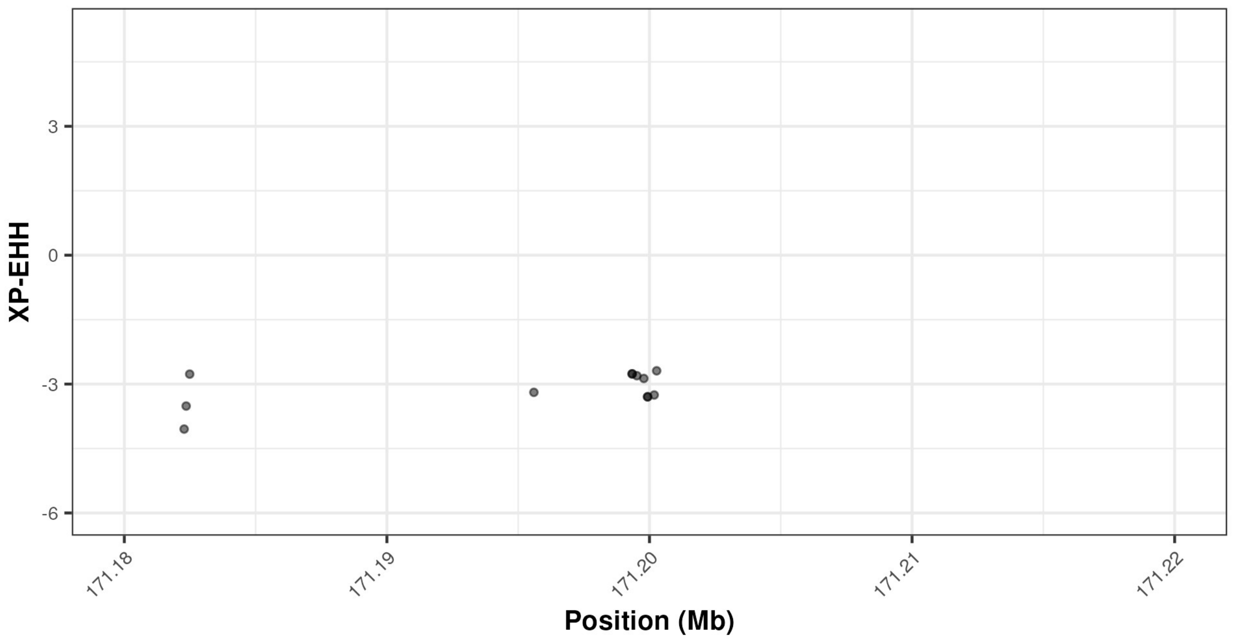

Selective sweep at chr 1:171.84-171.88 Mb with $|\text{XP-EHH}|_w = 3.46$

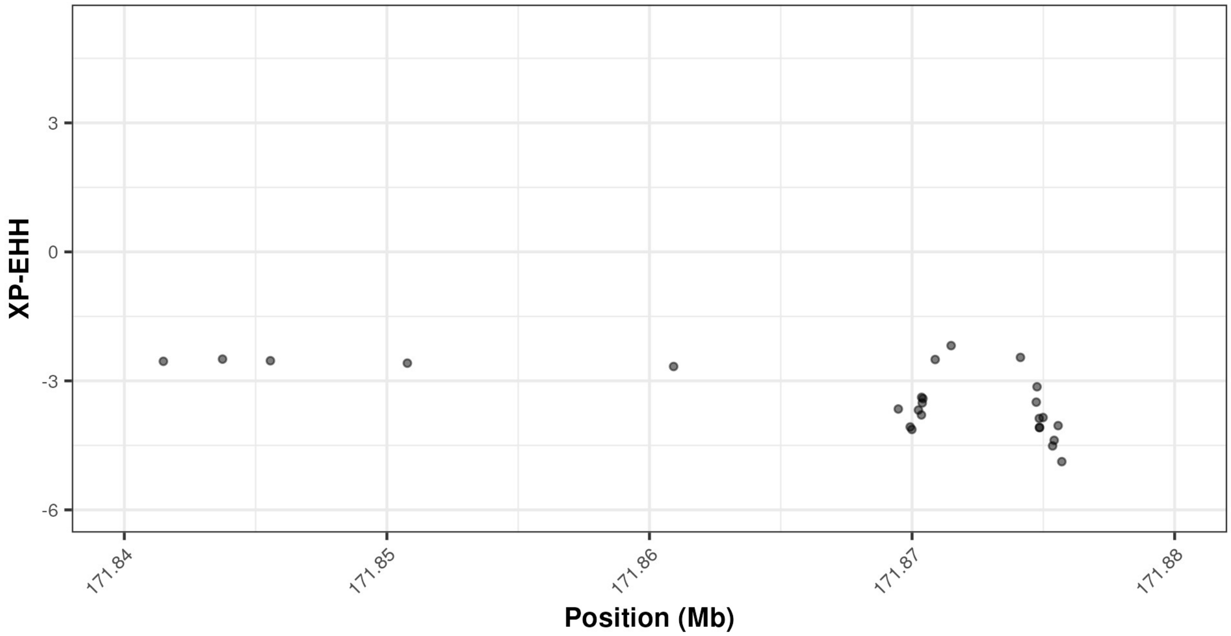

Selective sweep at chr 1:171.86-171.9 Mb with $|\text{XP-EHH}|_w = 3.48$

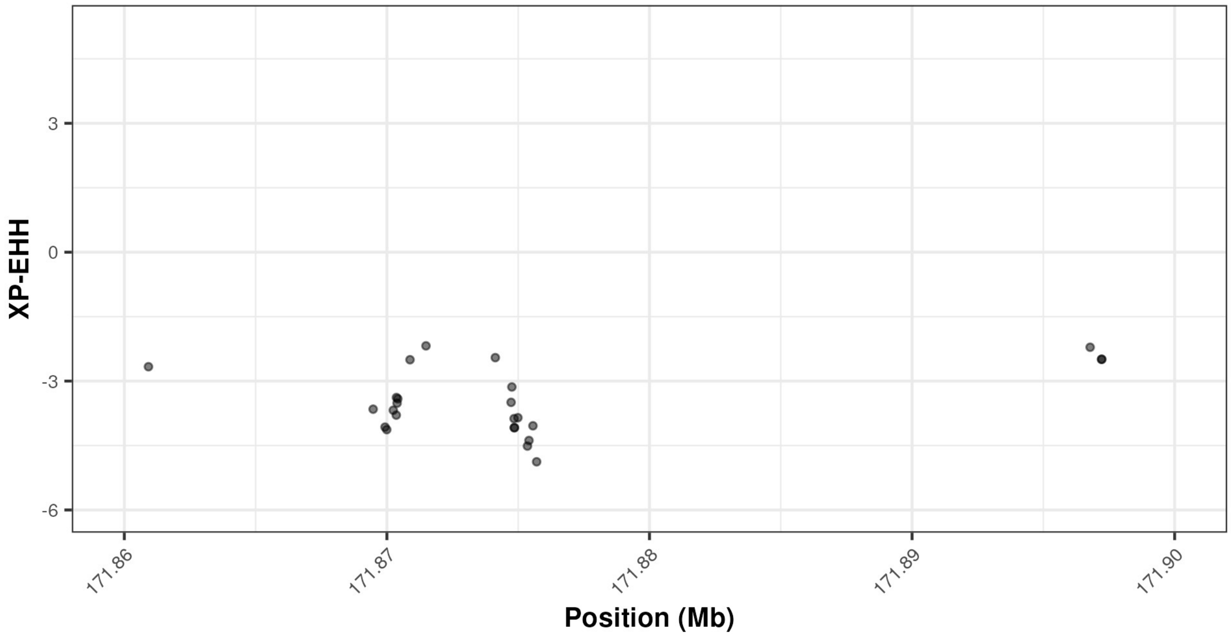

Selective sweep at chr 4:90.1-90.14 Mb with $|XP\text{-}EHH|_w = 3.27$

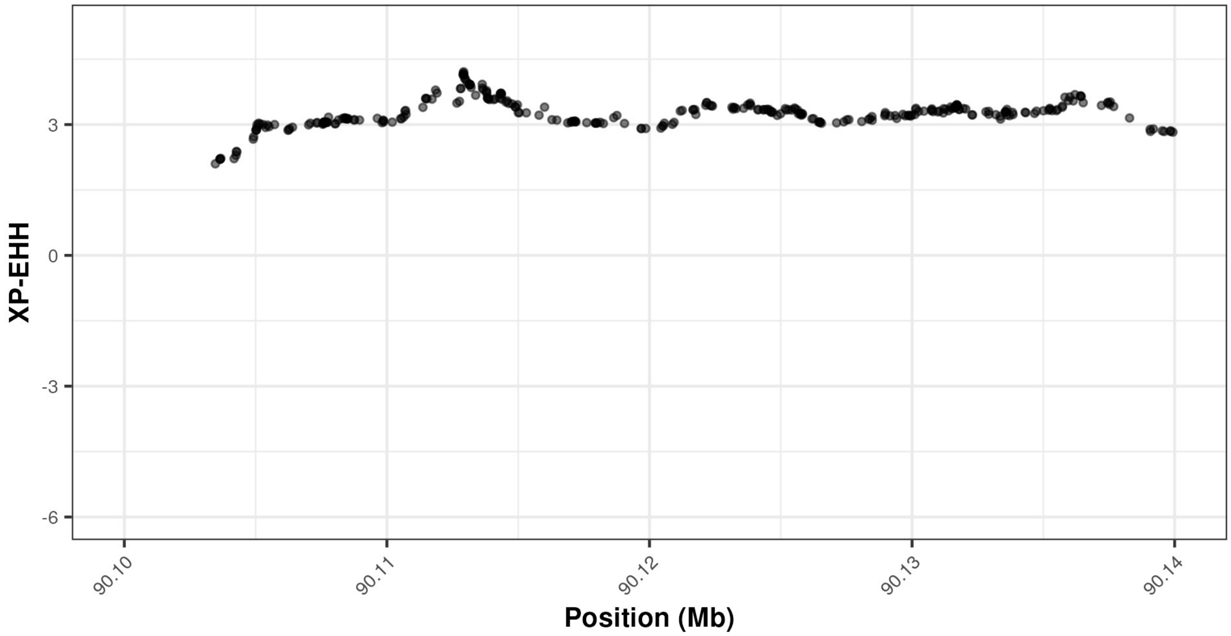

Selective sweep at chr 4:90.12-90.16 Mb with $|XP\text{-}EHH|_w = 3.28$

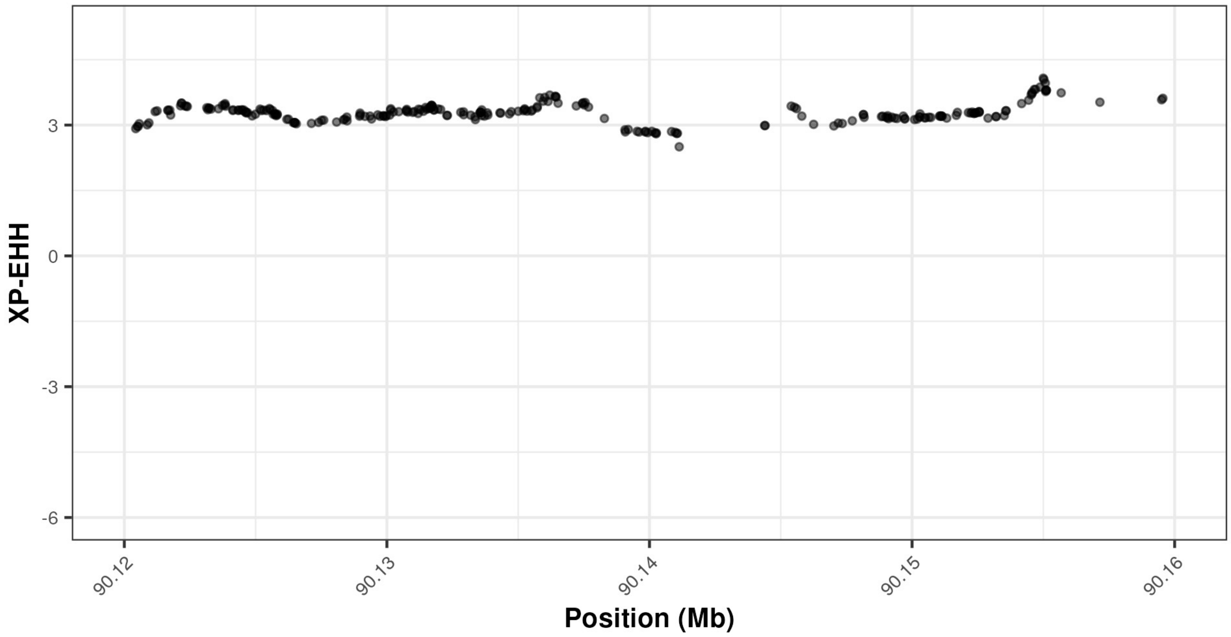

Selective sweep at chr 4:90.14-90.18 Mb with $|\text{XP-EHH}|_w = 3.28$

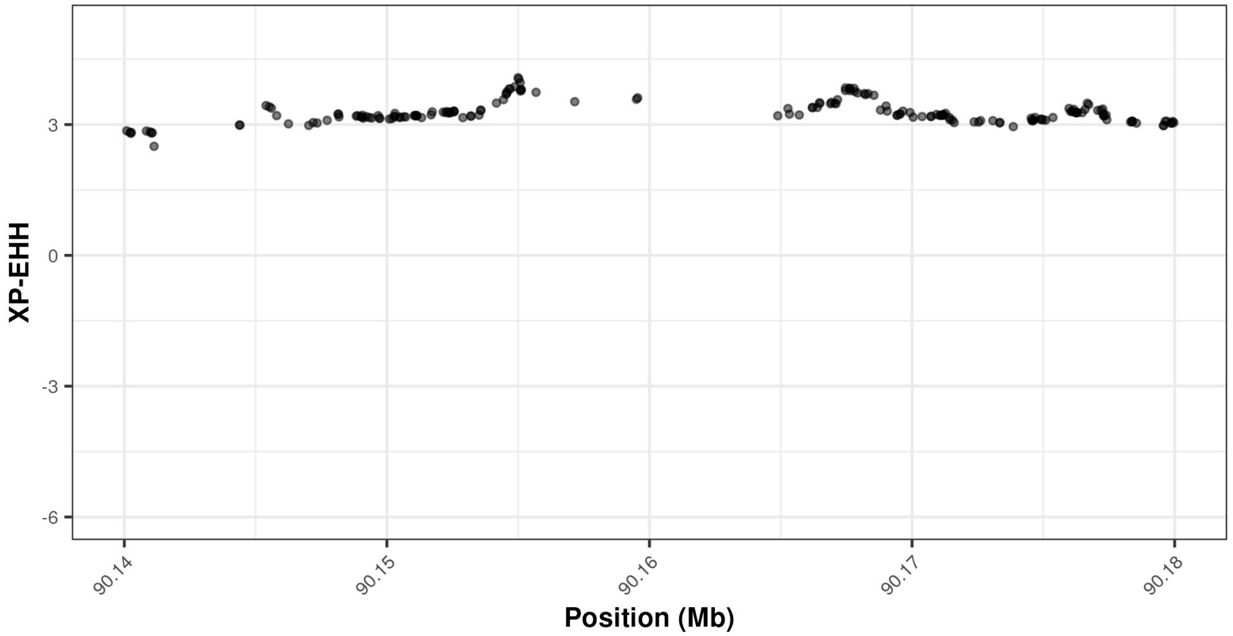

Selective sweep at chr 5:3.06-3.1 Mb with $|\text{XP-EHH}|_w = 3.98$

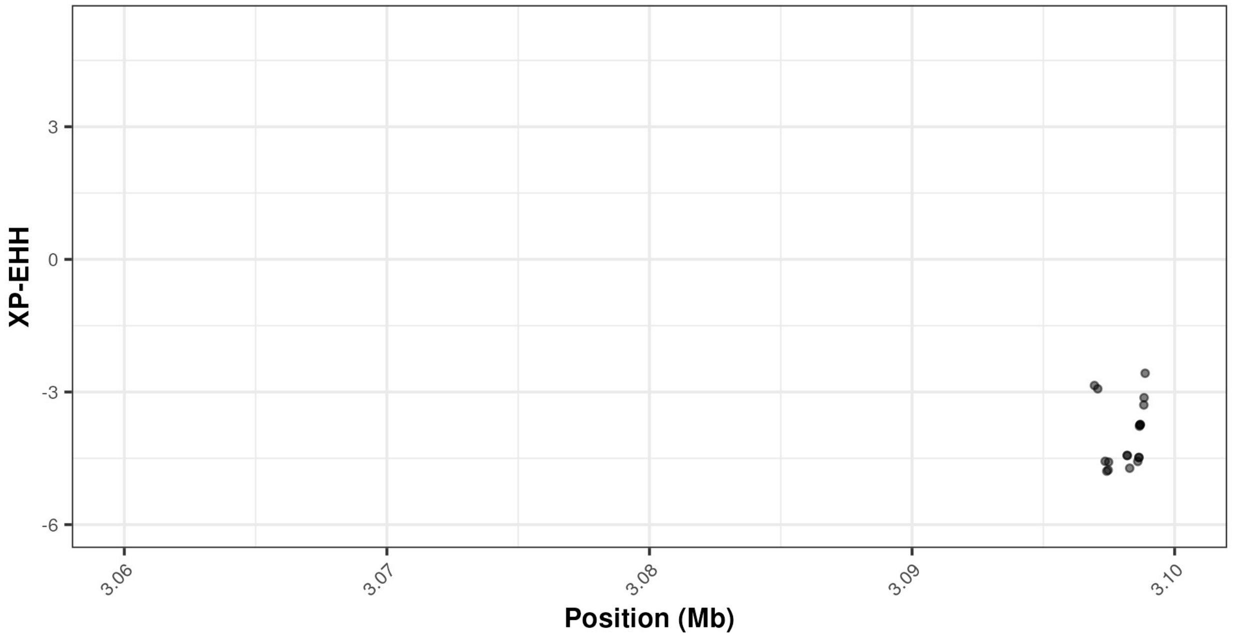

Selective sweep at chr 5:3.08-3.12 Mb with $|XP\text{-}EHH|_w = 3.98$

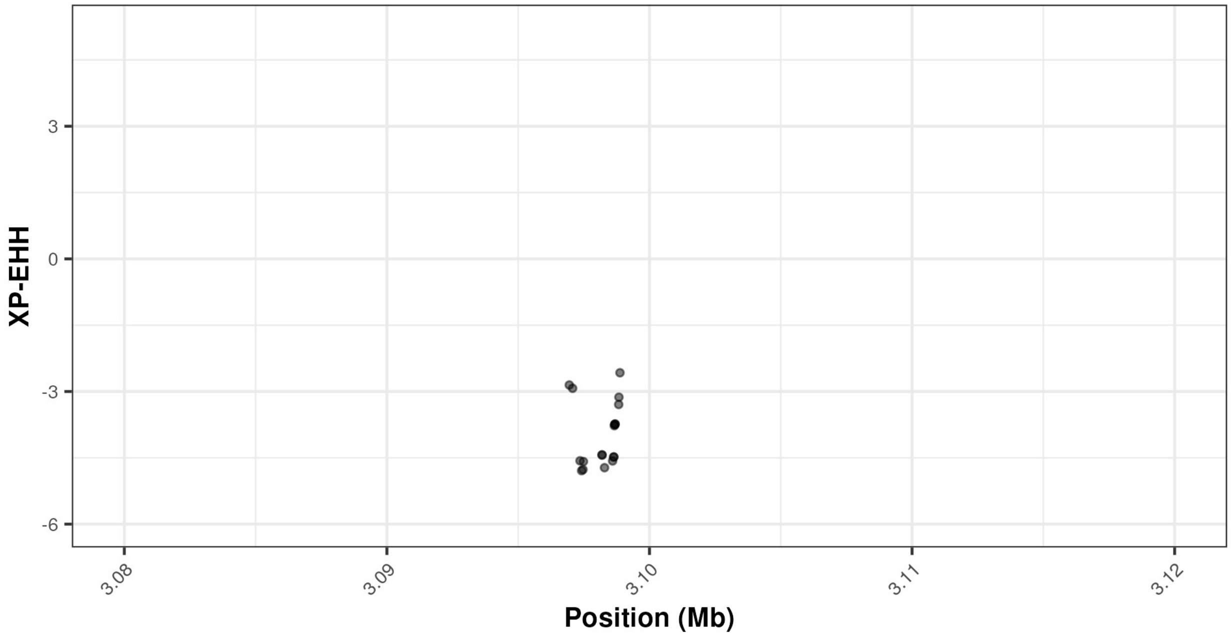

Selective sweep at chr 5:4.16-4.2 Mb with $|XP\text{-}EHH|_w = 3.1$

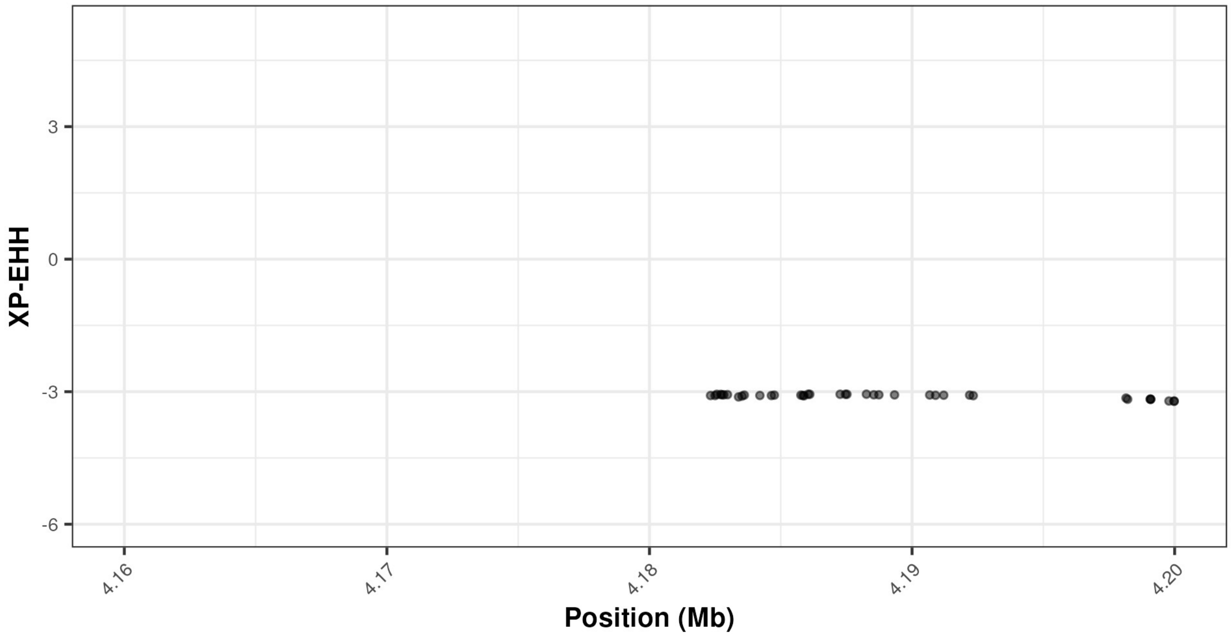

Selective sweep at chr 5:4.18-4.22 Mb with $|XP\text{-}EHH|_w = 3.11$

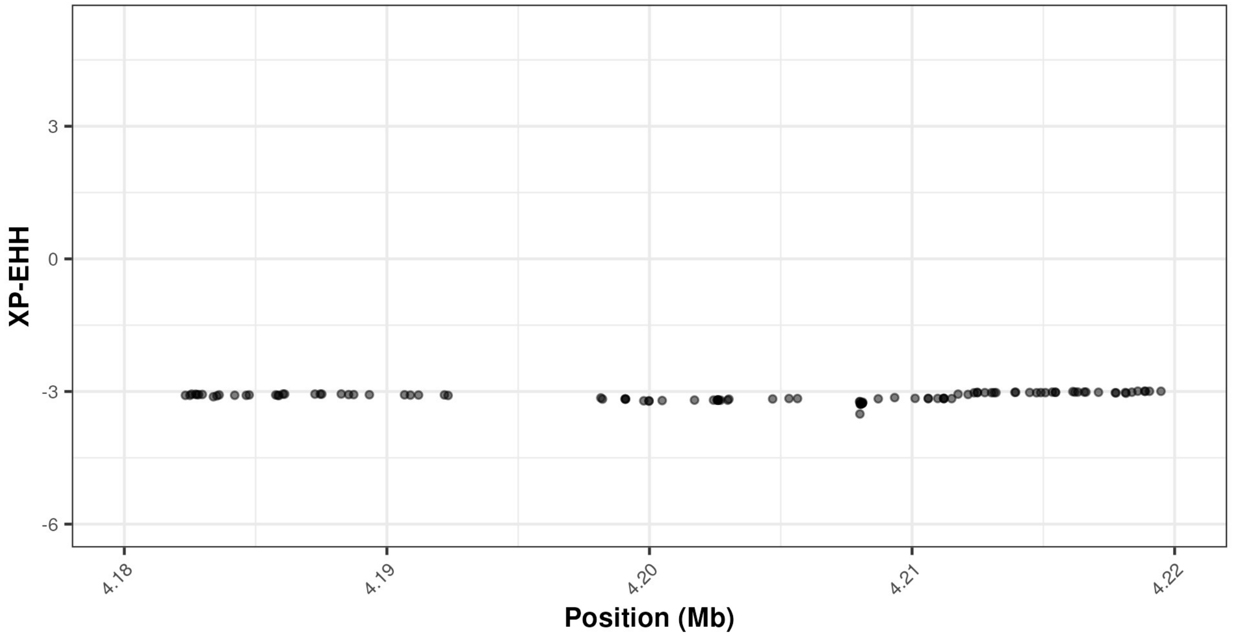

Selective sweep at chr 5:4.88-4.92 Mb with $|XP\text{-}EHH|_w = 3.09$

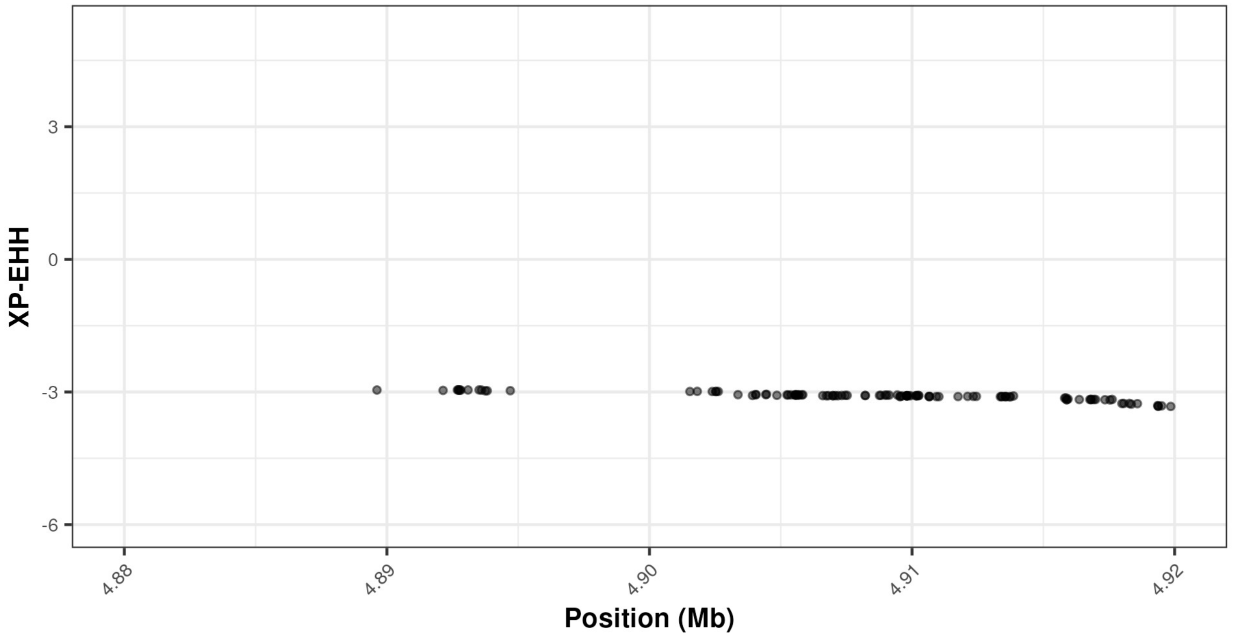

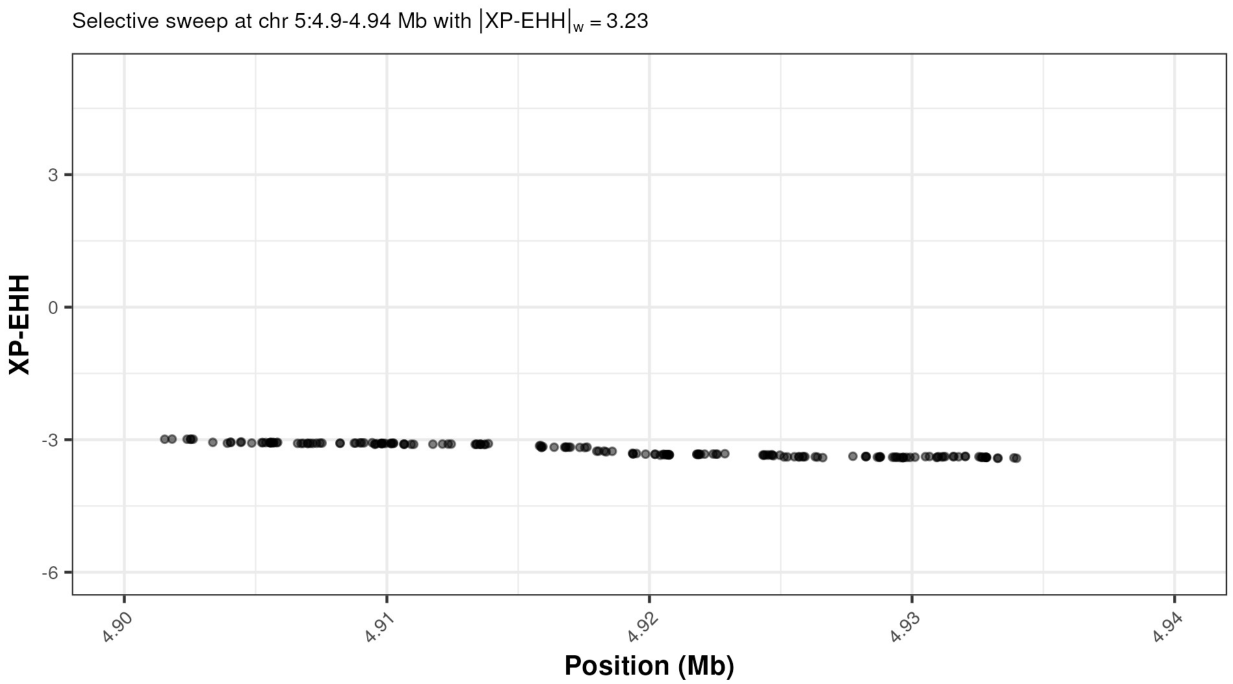
Selective sweep at chr 5:4.9-4.94 Mb with |XP-EHH|w = 3.23
XP-EHH
3
0
-3
-6
4.90
4.91
4.92
4.93
4.94
Position (Mb)

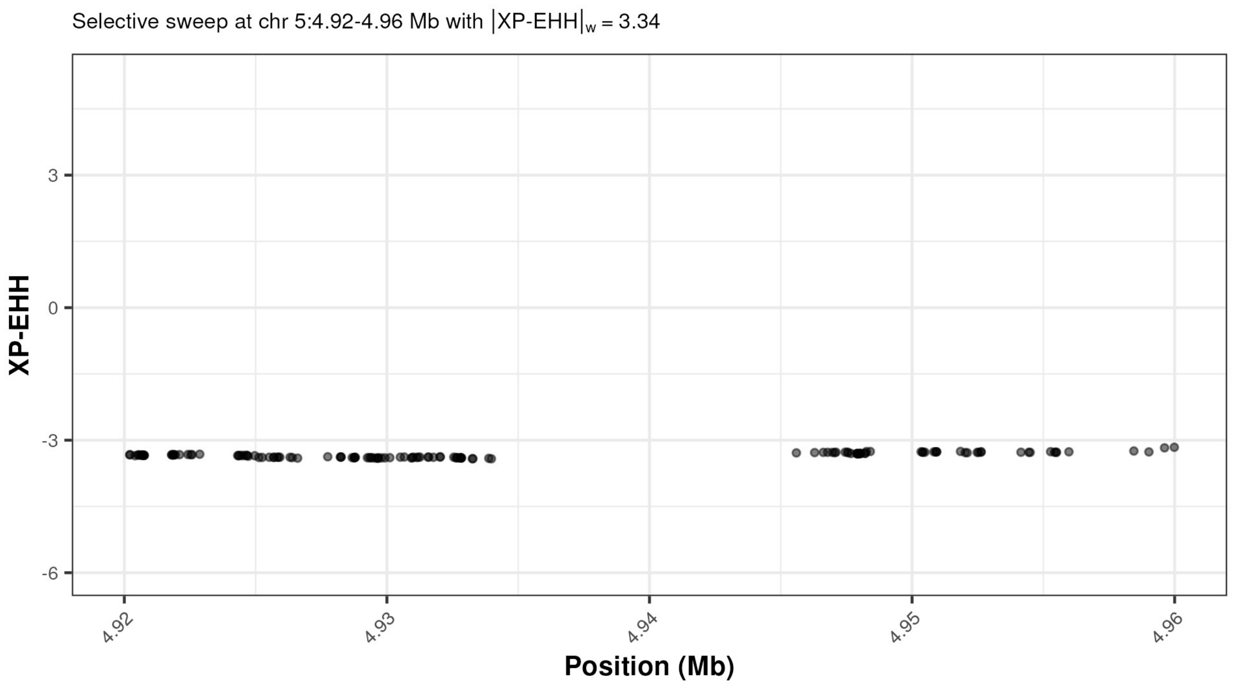
Selective sweep at chr 5:4.92-4.96 Mb with |XP-EHH|w = 3.34
XP-EHH
3
0
-3
-6
4.92
4.93
4.94
4.95
4.96
Position (Mb)

Selective sweep at chr 5:4.94-4.98 Mb with $|\text{XP-EHH}|_w = 3.23$

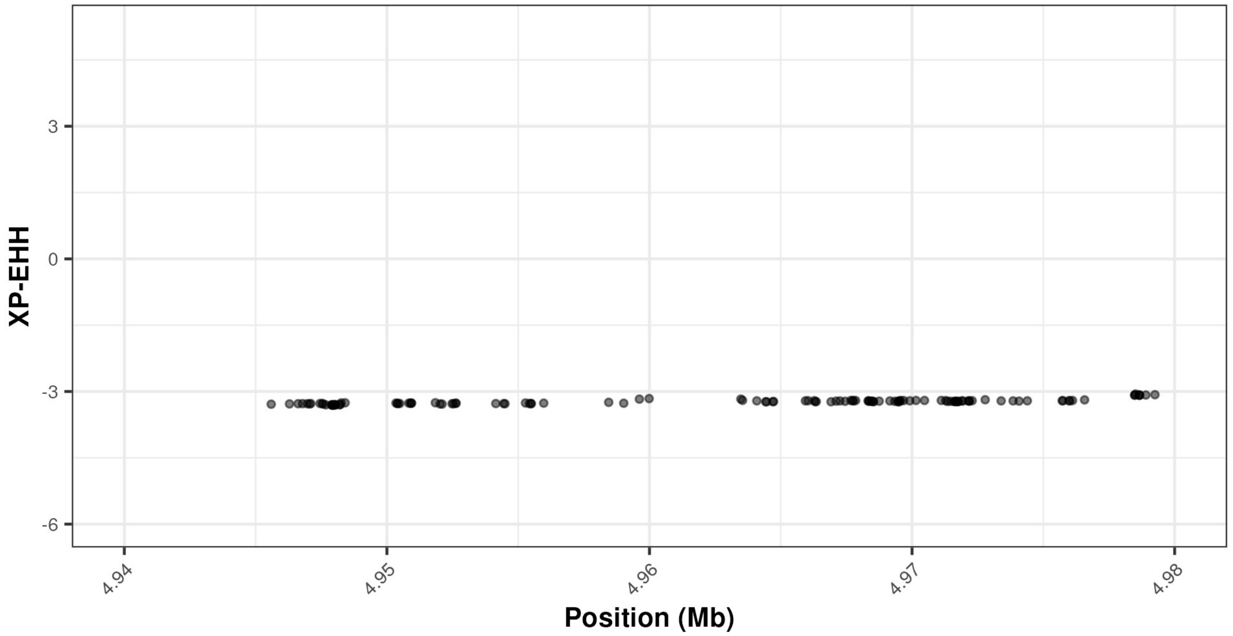

Selective sweep at chr 5:4.96-5 Mb with $|\text{XP-EHH}|_w = 3.17$

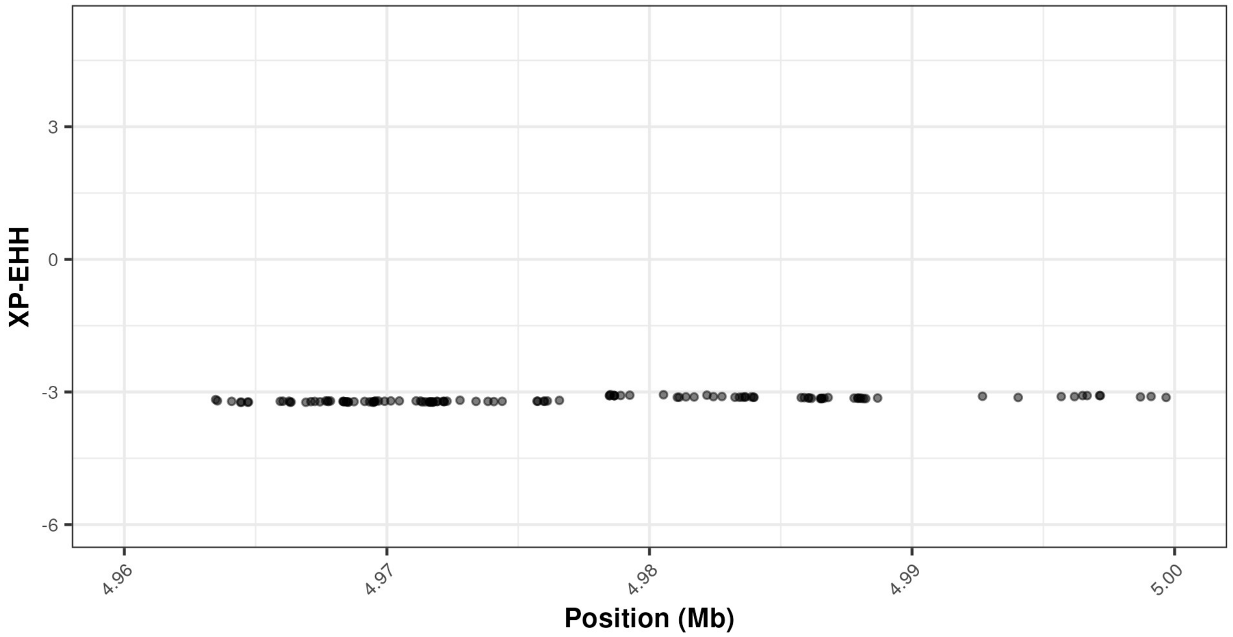

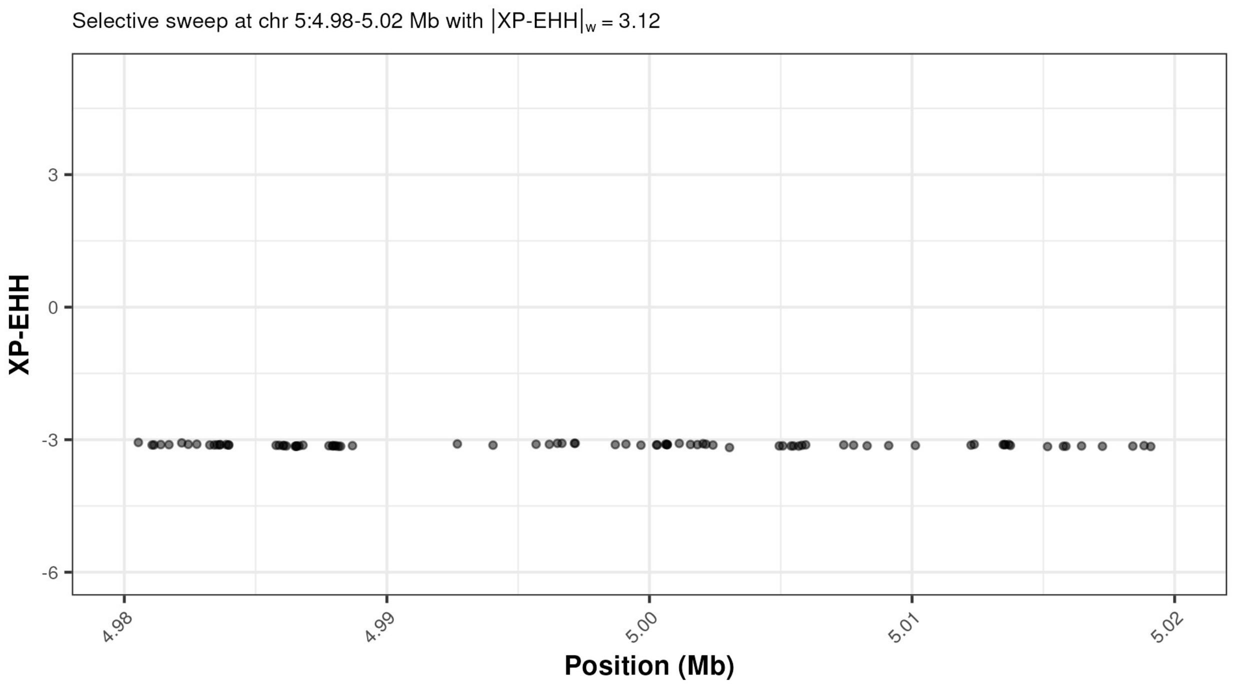
Selective sweep at chr 5:4.98-5.02 Mb with |XP-EHH|w = 3.12
XP-EHH
3
0
-3
-6
4.98
4.99
5.00
5.01
5.02
Position (Mb)

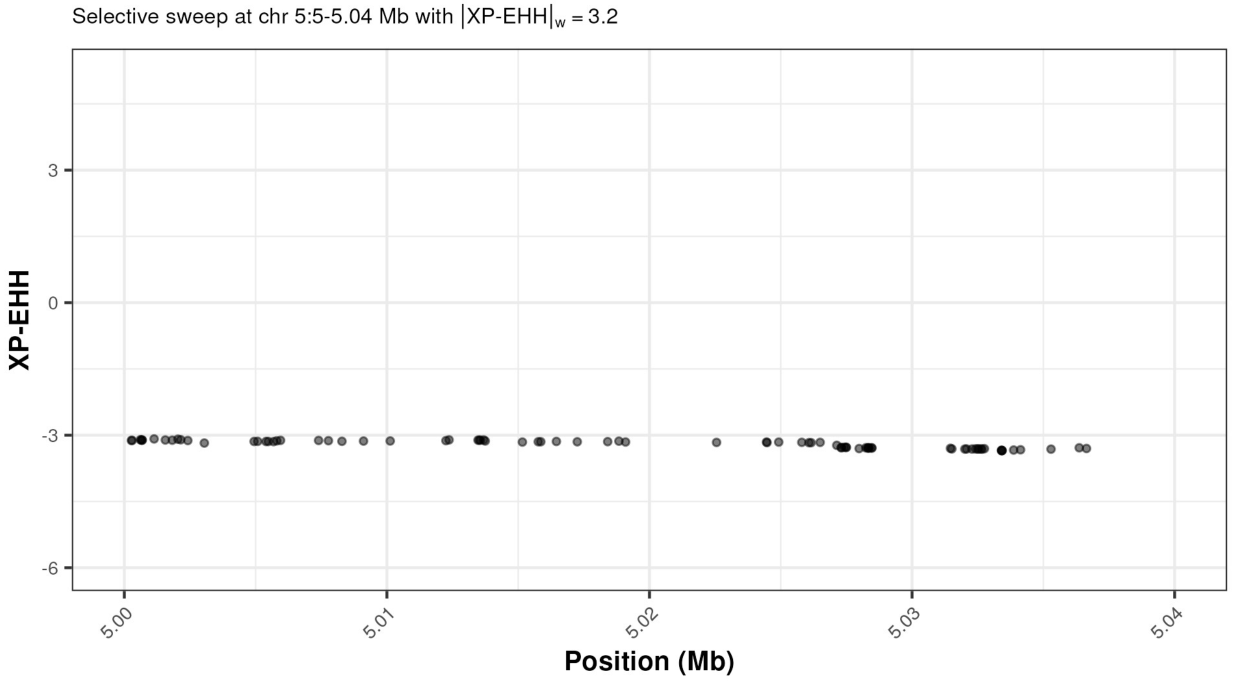
Selective sweep at chr 5:5-5.04 Mb with |XP-EHH|w = 3.2
XP-EHH
3
0
-3
-6
5.00
5.01
5.02
5.03
5.04
Position (Mb)

Selective sweep at chr 5:5.02-5.06 Mb with $|\text{XP-EHH}|_w = 3.37$

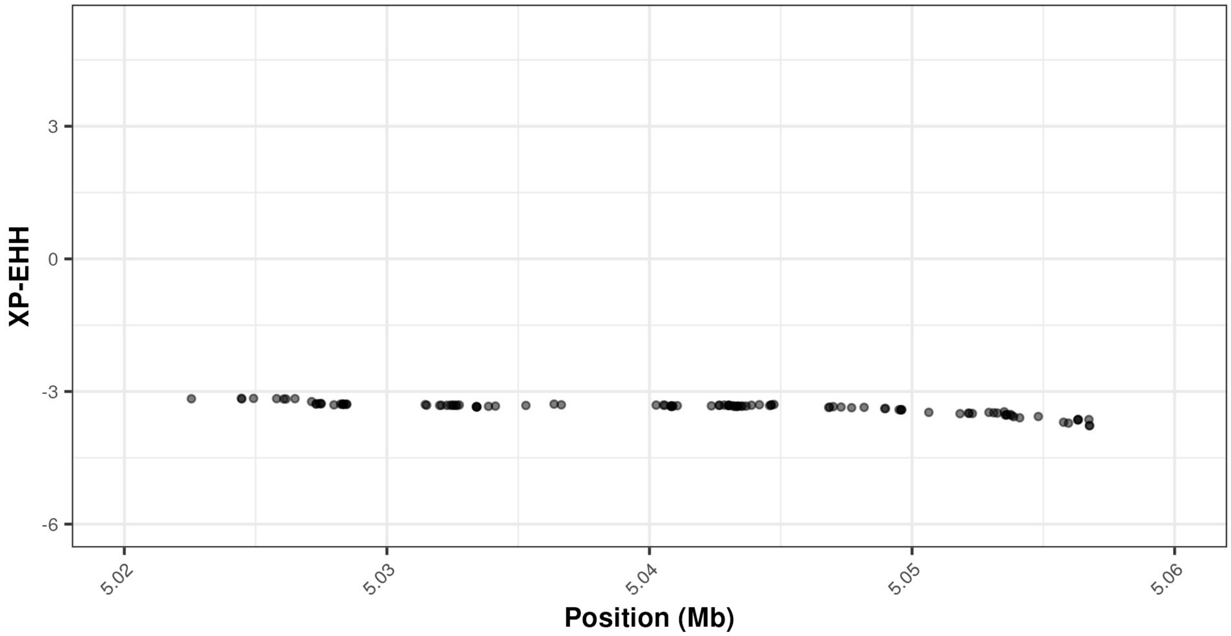

Selective sweep at chr 5:5.04-5.08 Mb with $|\text{XP-EHH}|_w = 3.47$

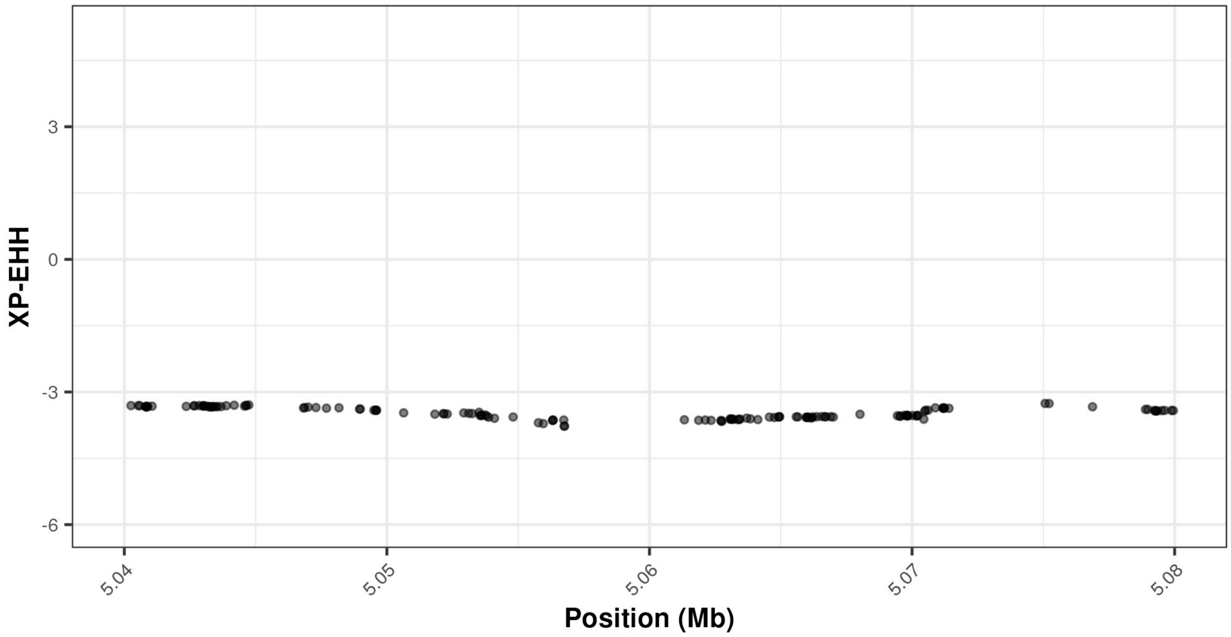

Selective sweep at chr 5:5.06-5.1 Mb with $|\text{XP-EHH}|_w = 3.48$

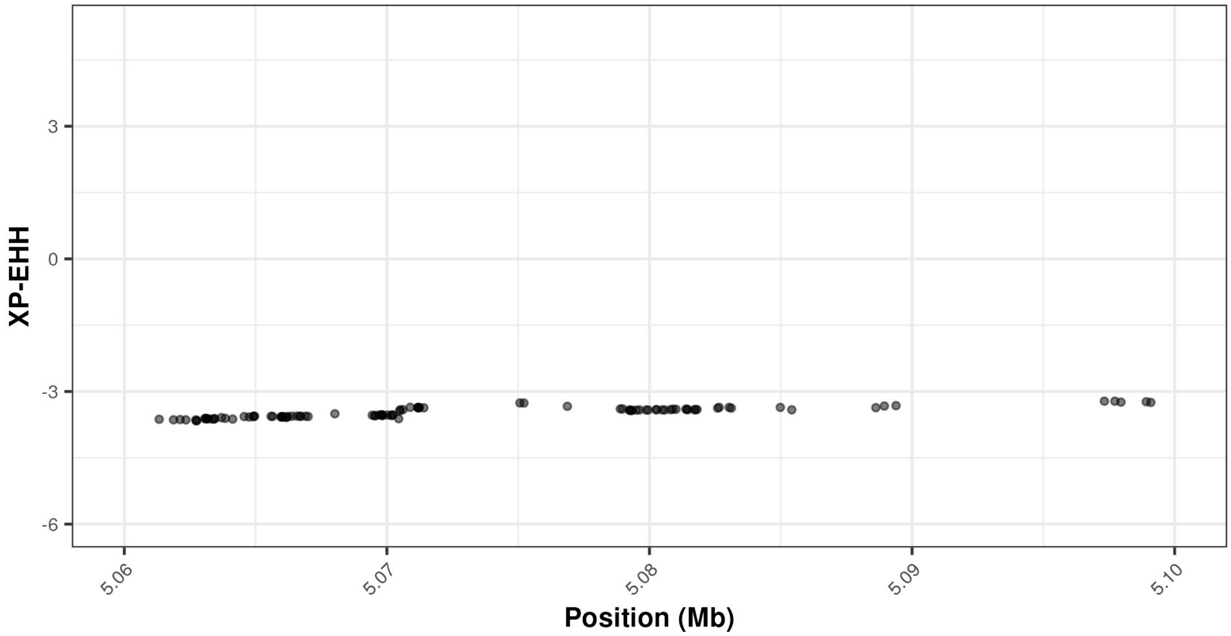

Selective sweep at chr 5:5.08-5.12 Mb with $|\text{XP-EHH}|_w = 3.3$

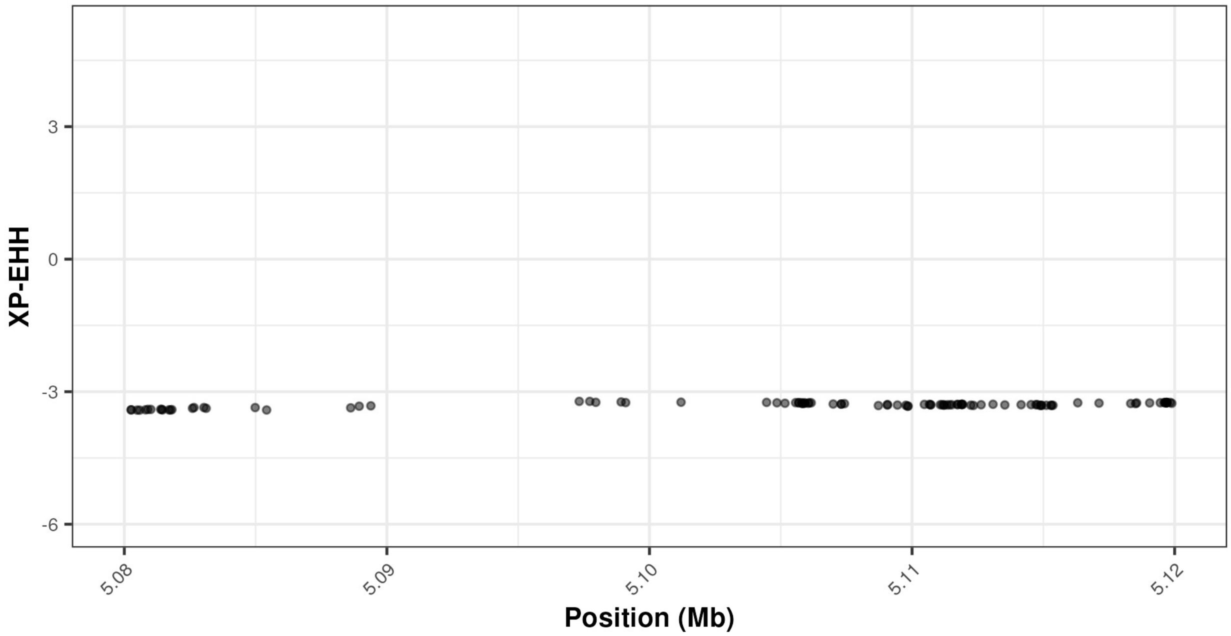

Selective sweep at chr 5:5.1-5.14 Mb with $|\text{XP-EHH}|_w = 3.28$

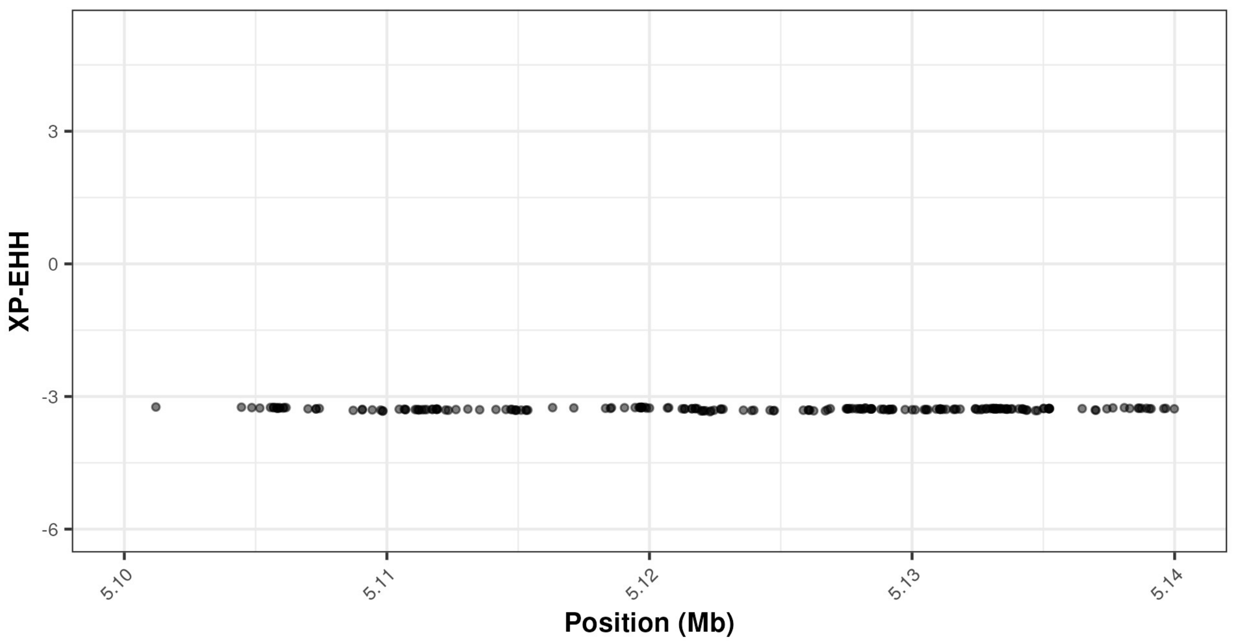

Selective sweep at chr 5:5.12-5.16 Mb with $|\text{XP-EHH}|_w = 3.24$

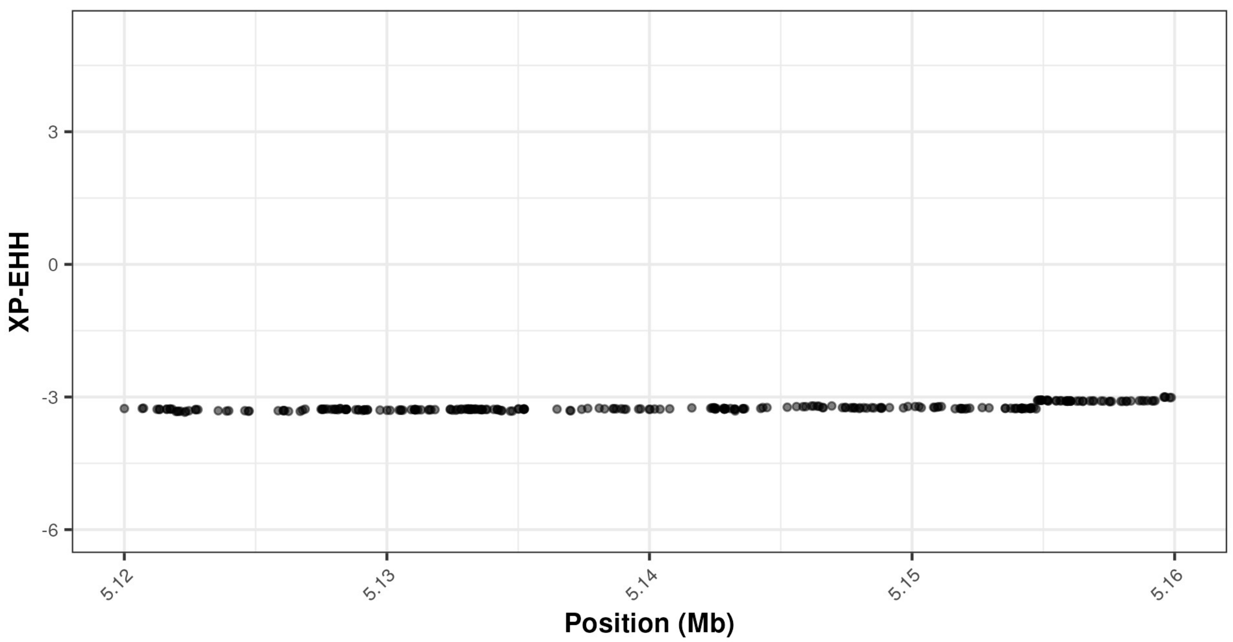

Selective sweep at chr 5:5.14-5.18 Mb with $|\text{XP-EHH}|_w = 3.12$

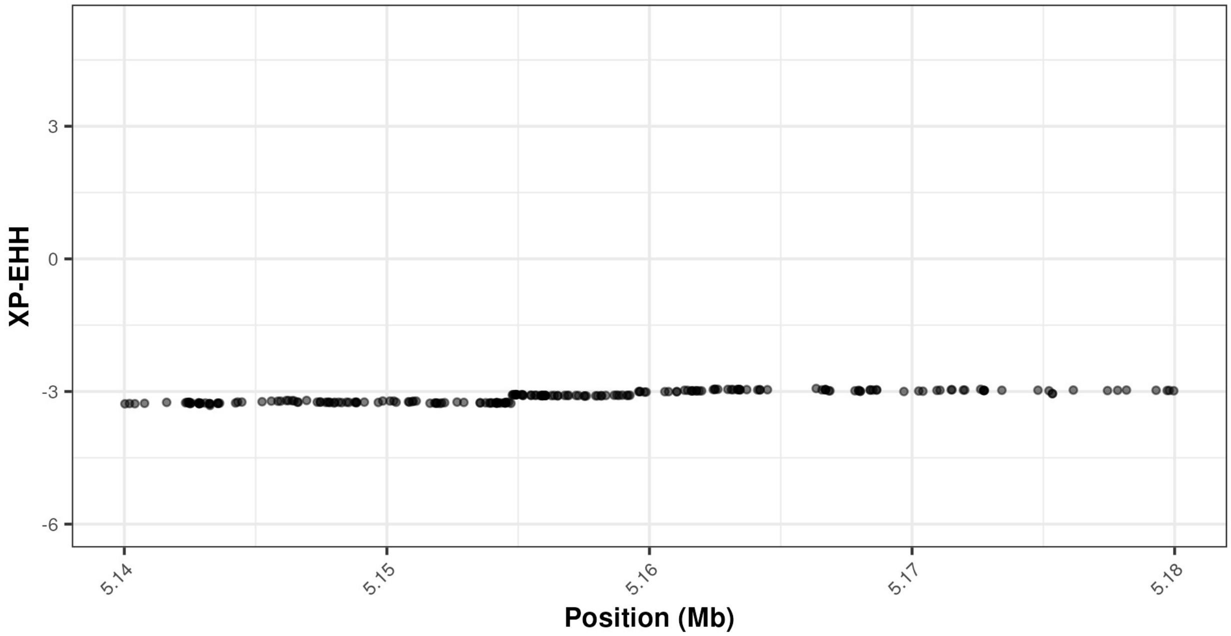

Selective sweep at chr 5:5.22-5.26 Mb with $|\text{XP-EHH}|_w = 3.13$

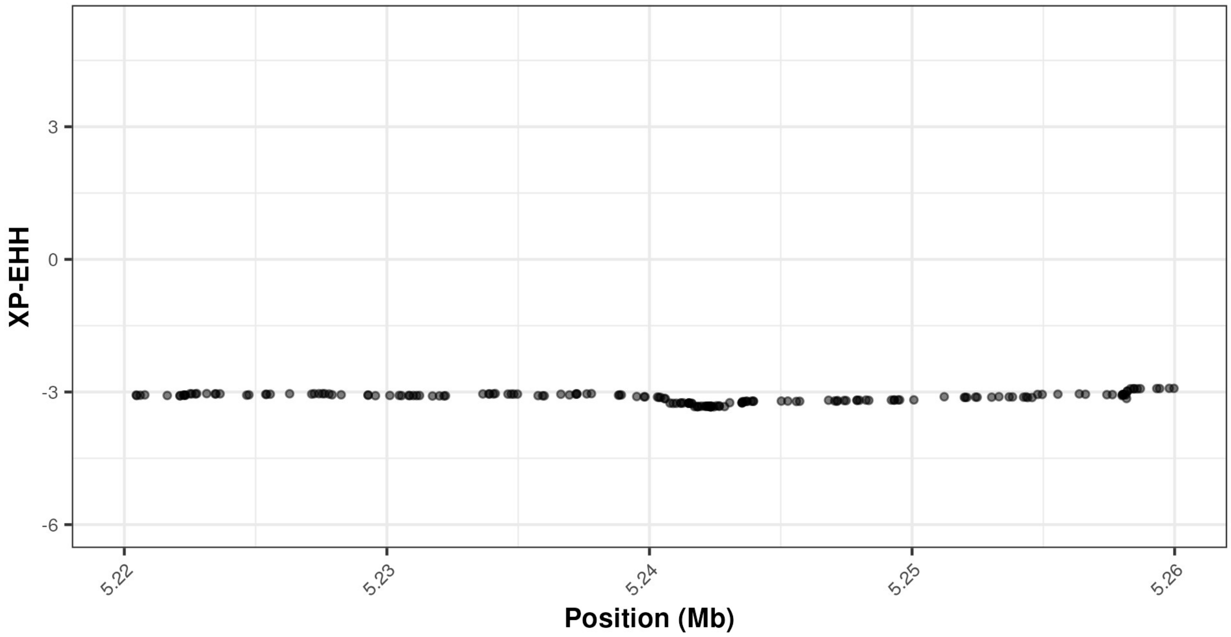

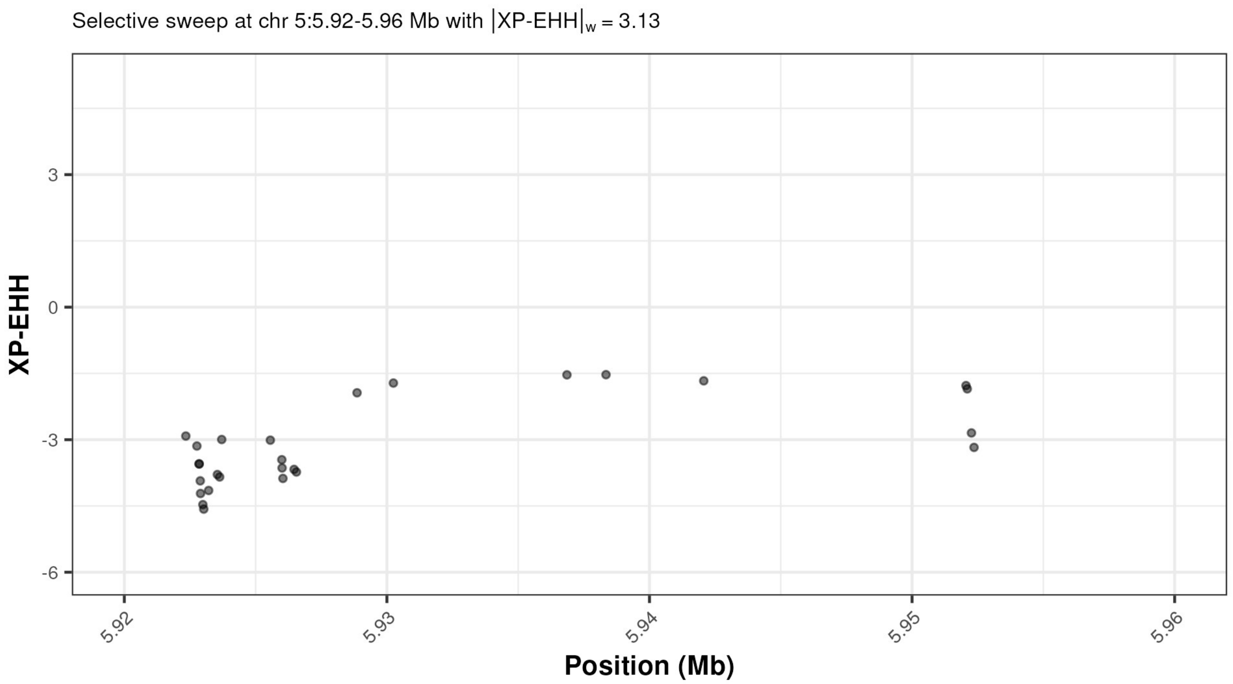
Selective sweep at chr 5:5.92-5.96 Mb with |XP-EHH|w = 3.13
3
0
-3
-6
XP-EHH
5.92
5.93
5.94
5.95
5.96
Position (Mb)

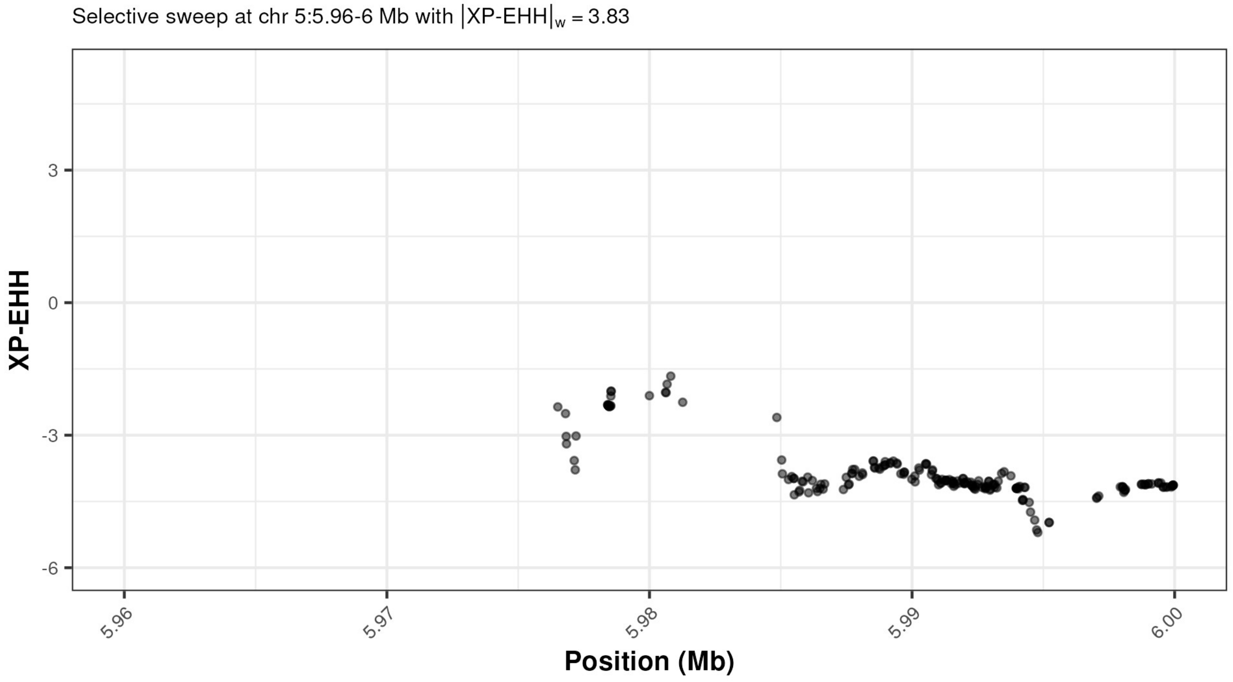
Selective sweep at chr 5:5.96-6 Mb with |XP-EHH|w = 3.83
3
0
-3
-6
XP-EHH
5.96
5.97
5.98
5.99
6.00
Position (Mb)

Selective sweep at chr 5:5.98-6.02 Mb with $|XP\text{-}EHH|_w = 4.05$

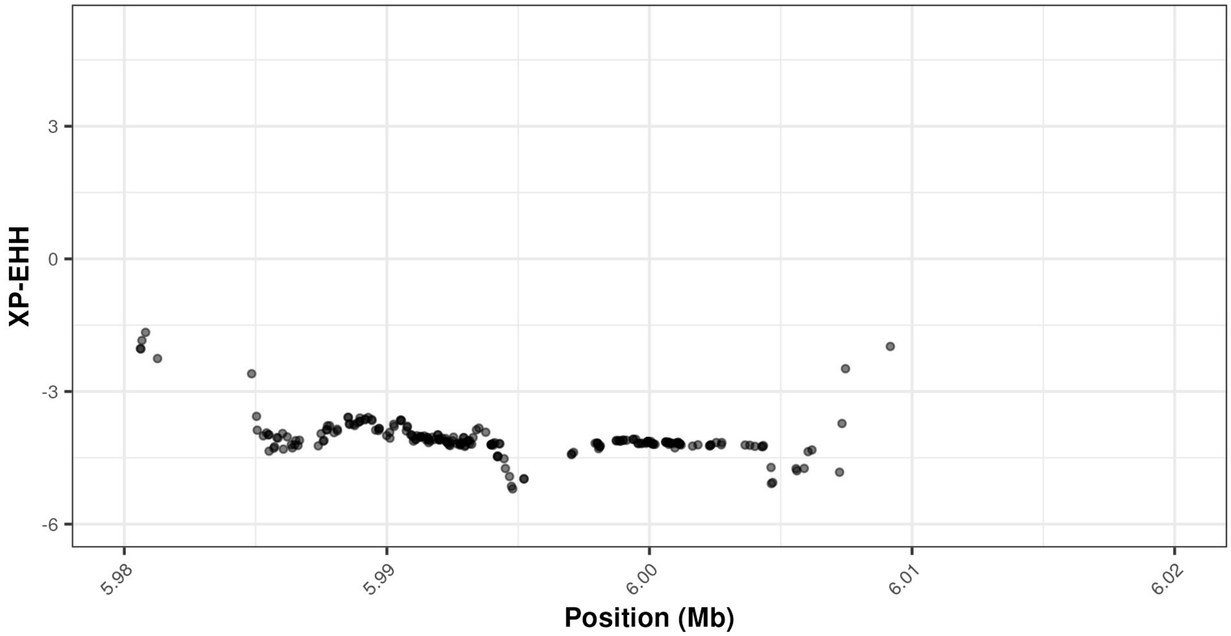

Selective sweep at chr 5:6-6.04 Mb with $|XP\text{-}EHH|_w = 4.2$

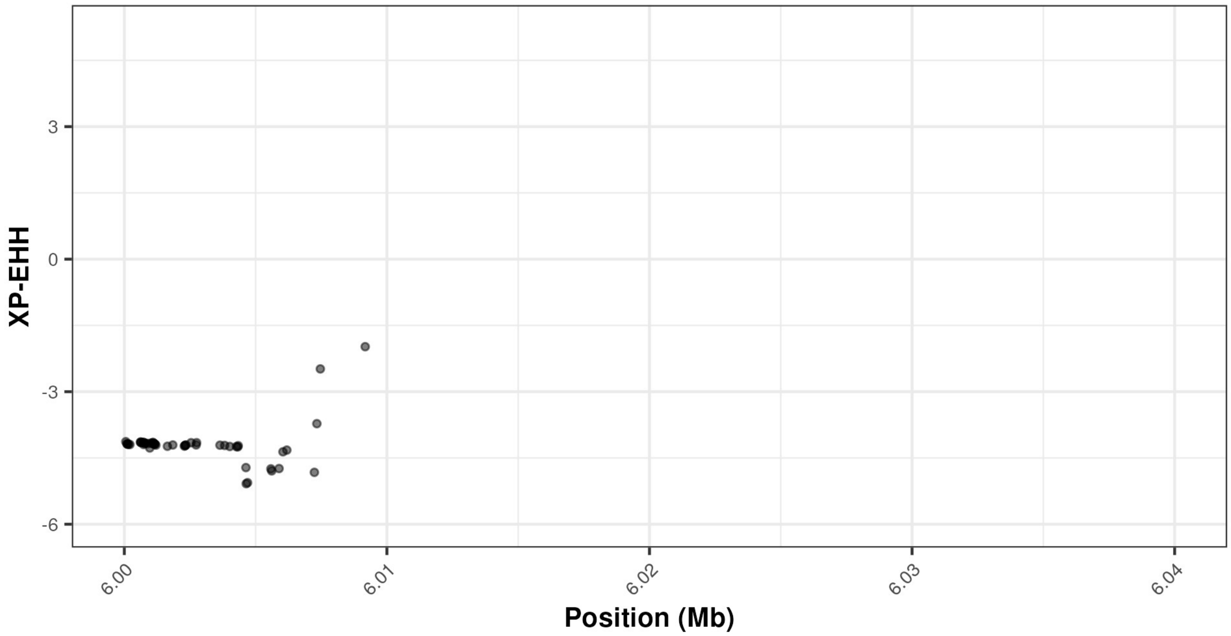

Selective sweep at chr 5:6.52-6.56 Mb with $|\text{XP-EHH}|_w = 3.42$

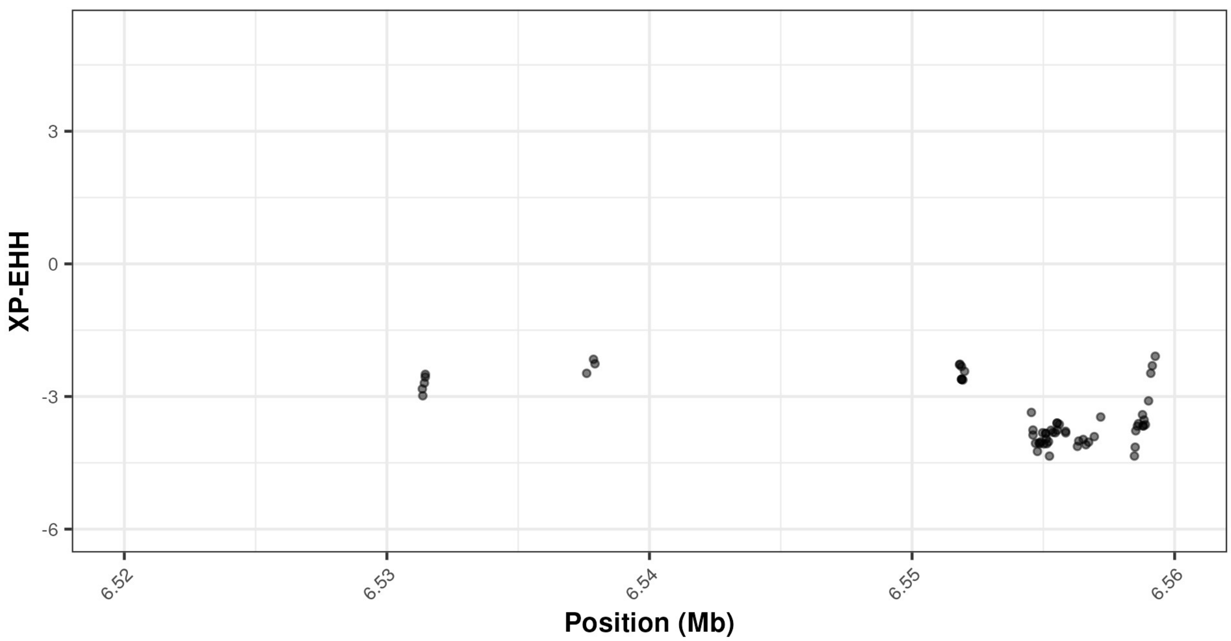

Selective sweep at chr 5:6.6-6.64 Mb with $|\text{XP-EHH}|_w = 3.35$

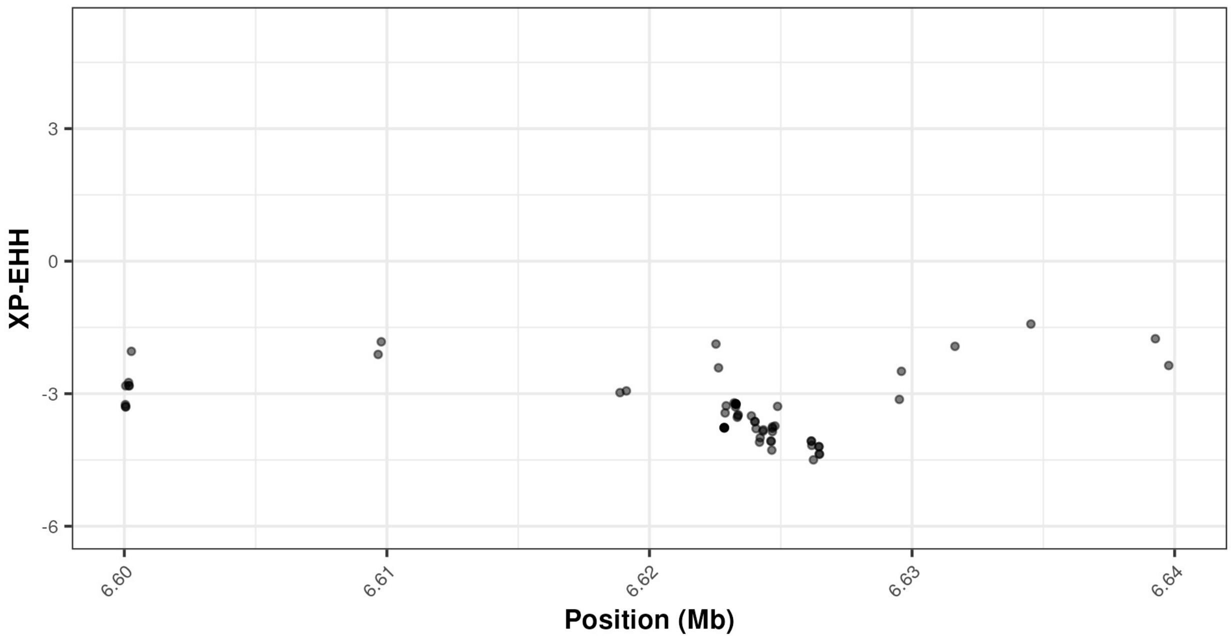

Selective sweep at chr 5:7.34-7.38 Mb with $|\text{XP-EHH}|_w = 3.08$

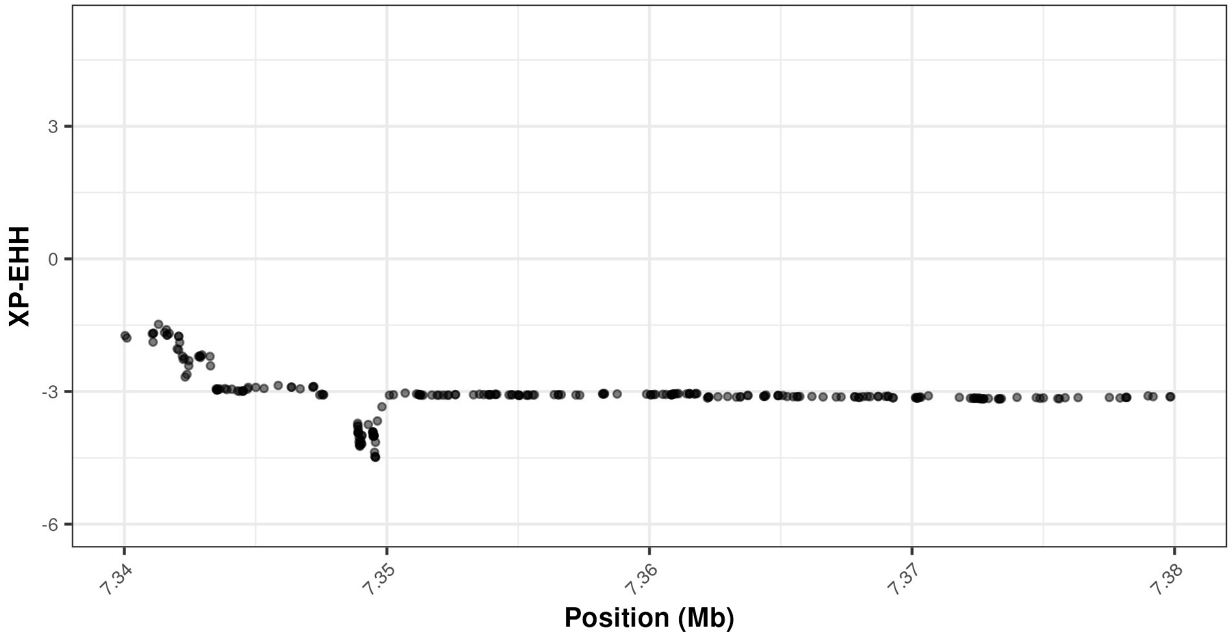

Selective sweep at chr 5:7.36-7.4 Mb with $|\text{XP-EHH}|_w = 3.13$

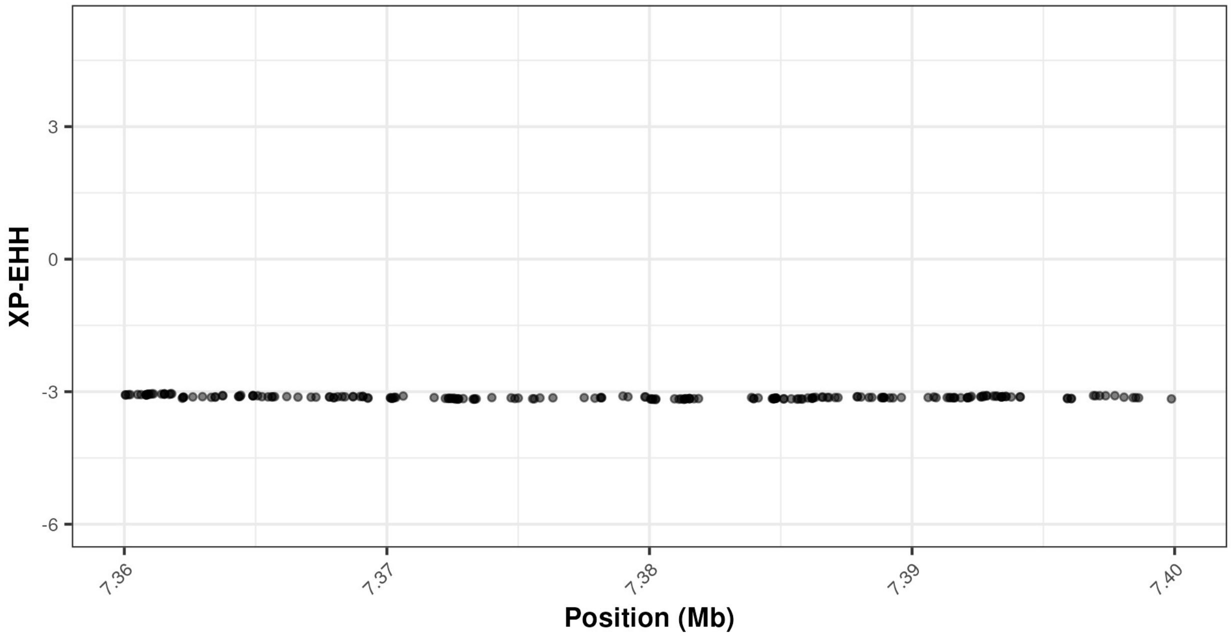

Selective sweep at chr 5:7.38-7.42 Mb with $|\text{XP-EHH}|_w = 3.14$

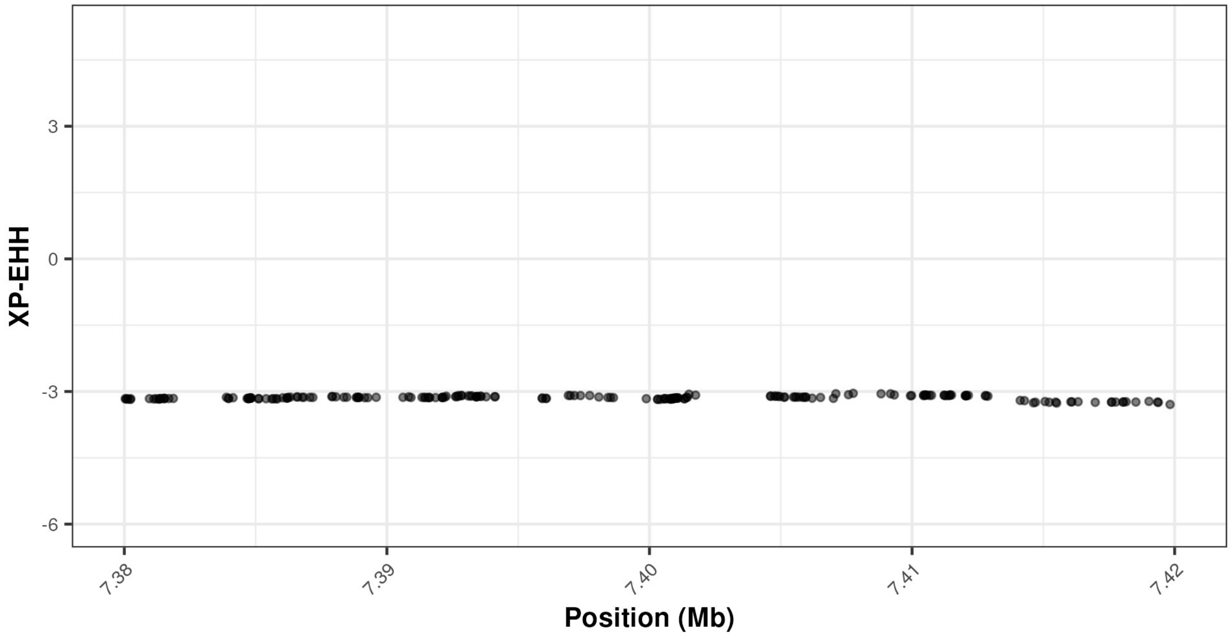

Selective sweep at chr 5:7.4-7.44 Mb with $|\text{XP-EHH}|_w = 3.27$

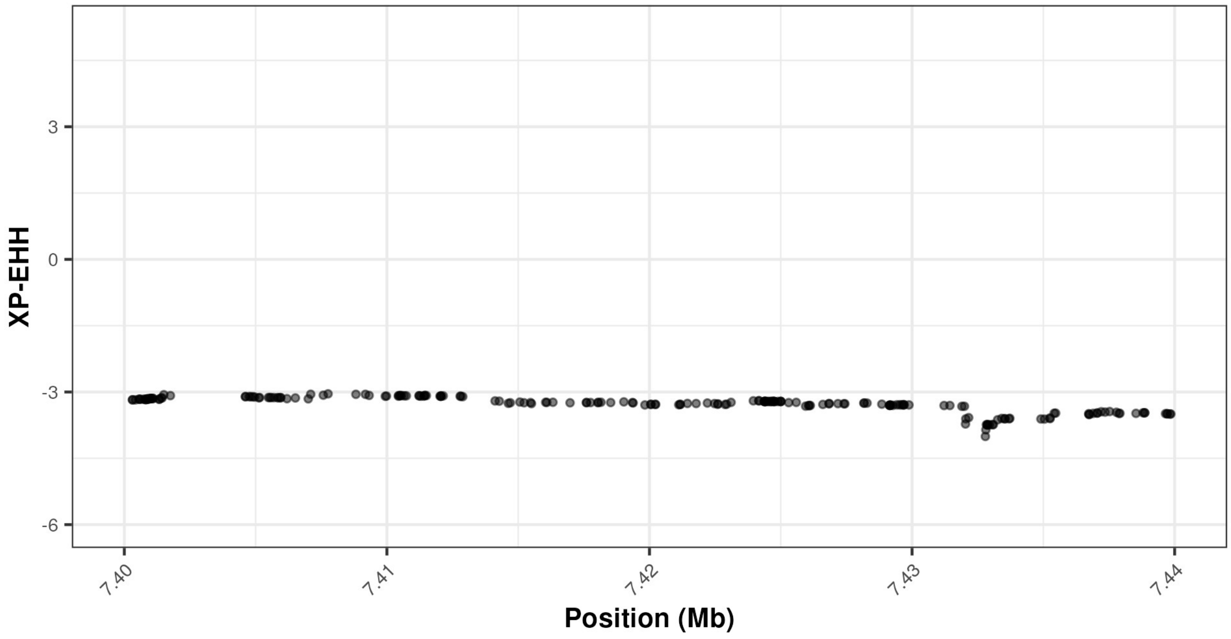

Selective sweep at chr 5:7.42-7.46 Mb with $|XP\text{-}EHH|_w = 3.43$

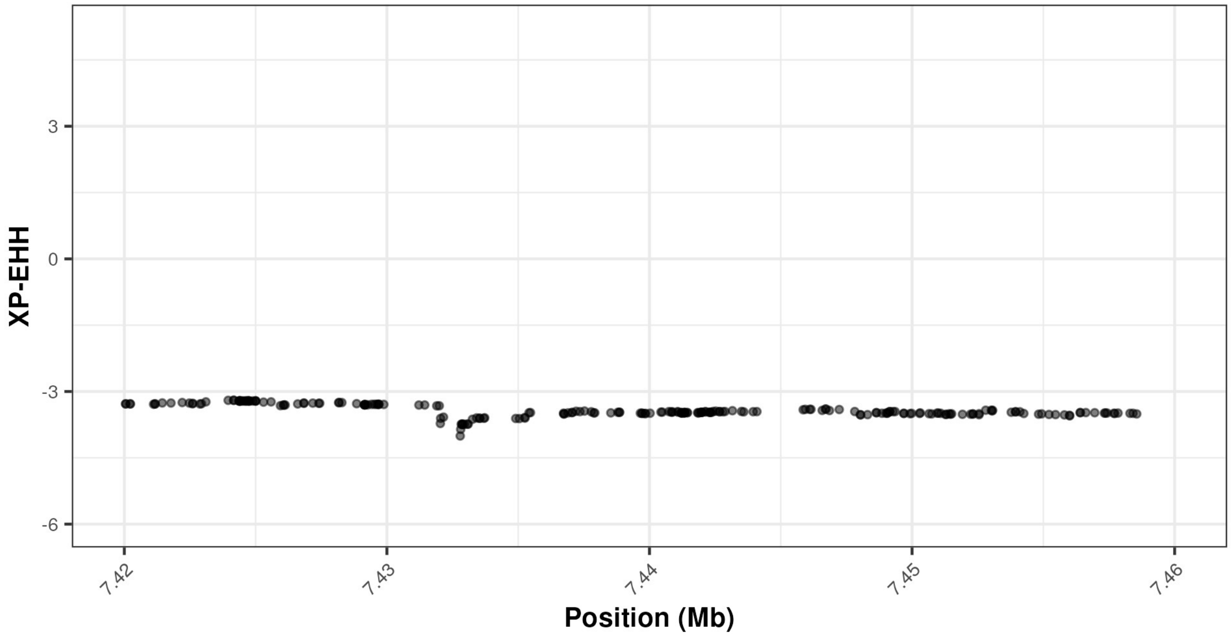

Selective sweep at chr 5:7.44-7.48 Mb with $|XP\text{-}EHH|_w = 3.48$

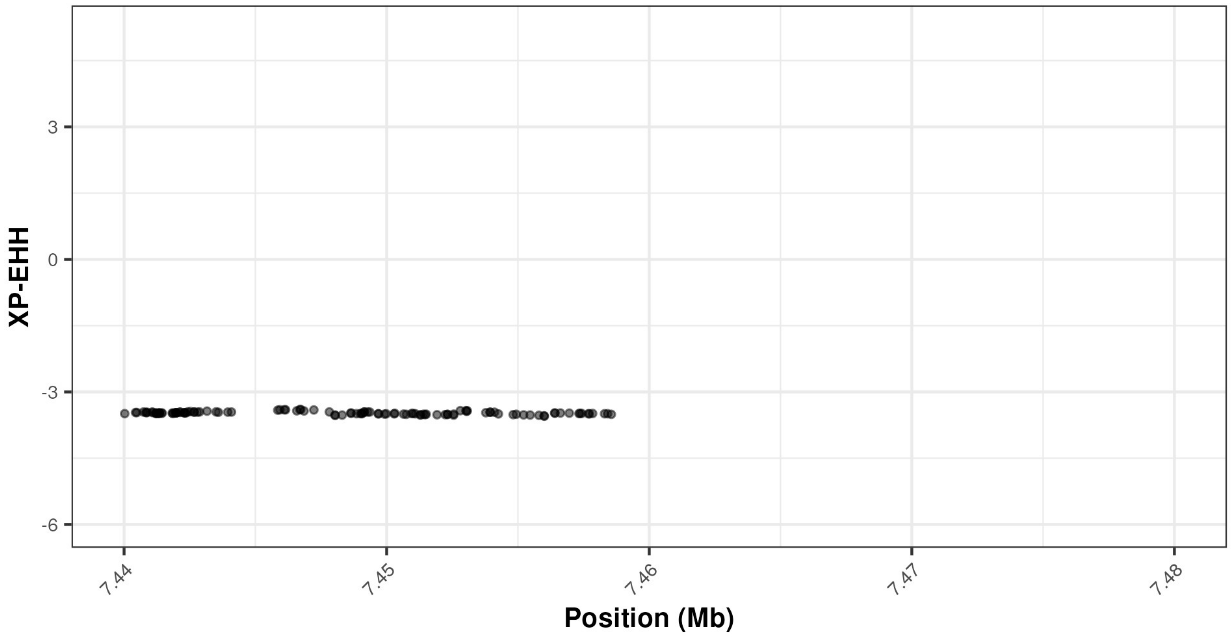

Selective sweep at chr 5:7.74-7.78 Mb with $|\text{XP-EHH}|_w = 3.35$

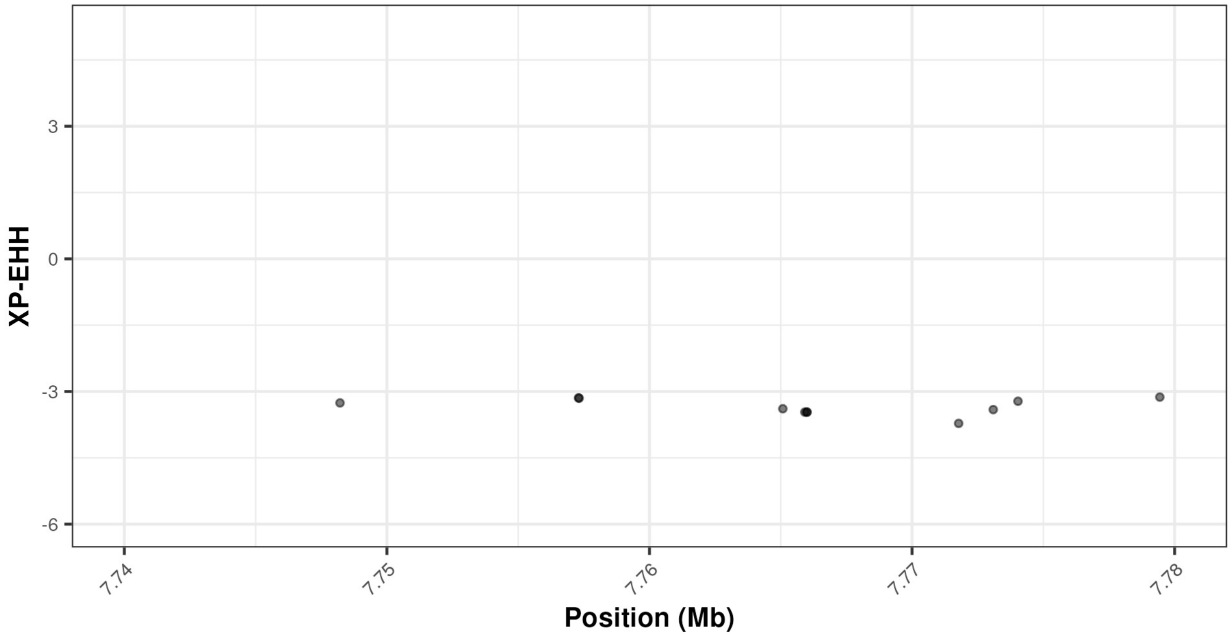

Selective sweep at chr 6:1.06-1.1 Mb with $|\text{XP-EHH}|_w = 3.47$

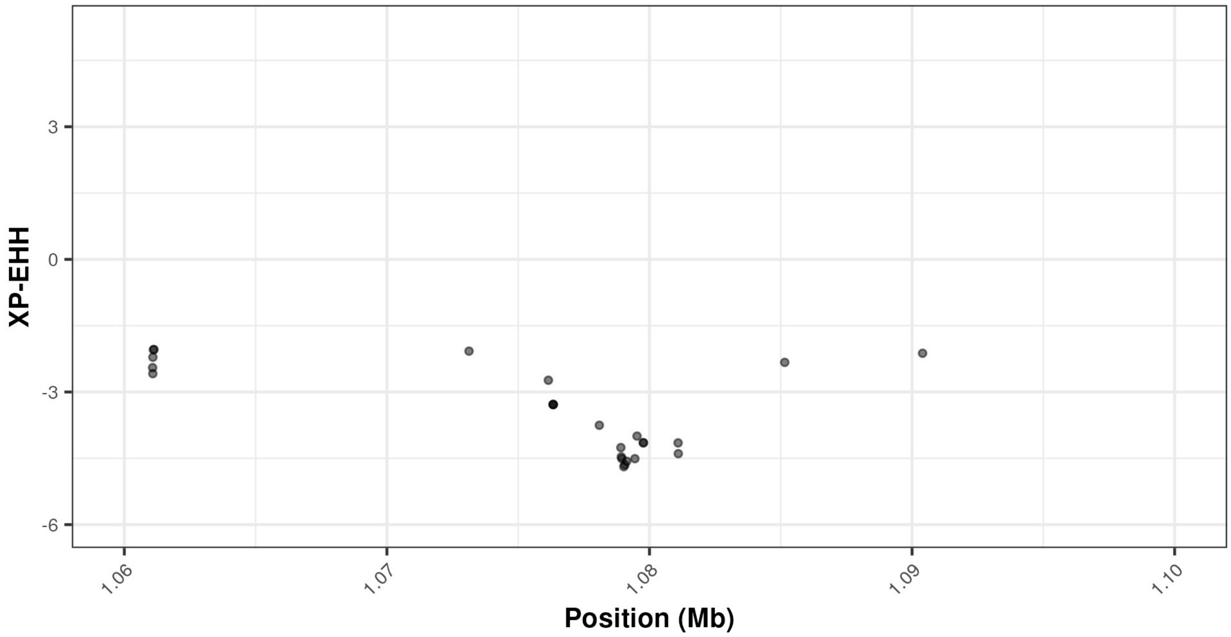

Selective sweep at chr 6:21.96-22 Mb with $|XP\text{-}EHH|_w = 3.1$

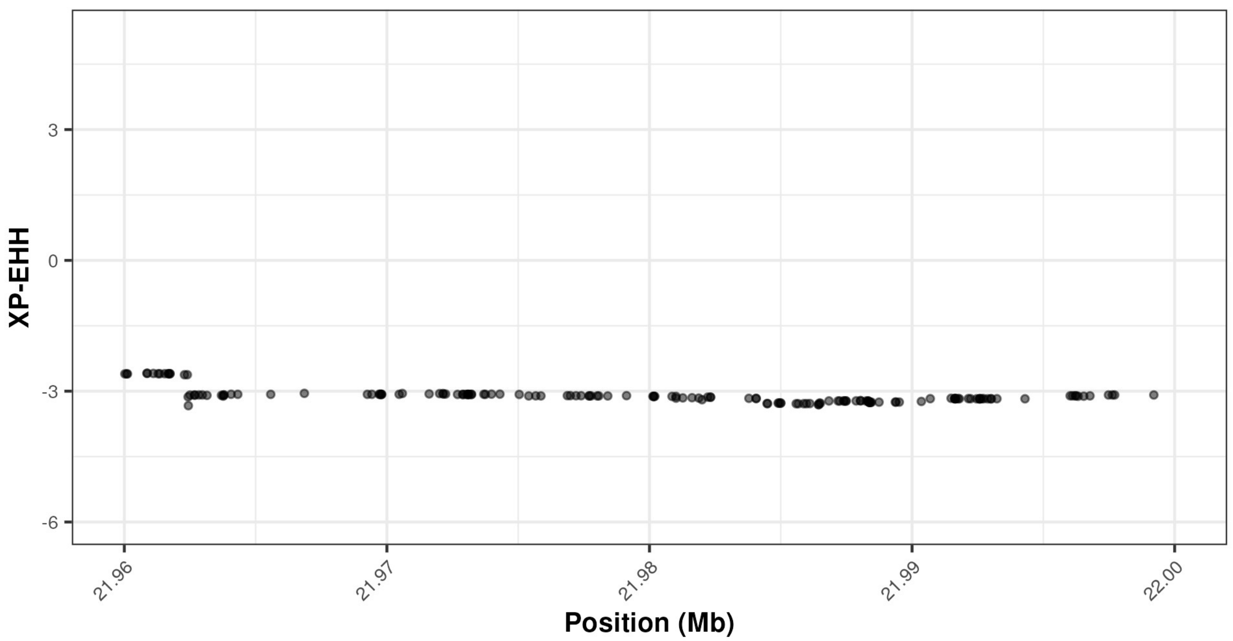

Selective sweep at chr 6:21.98-22.02 Mb with $|XP\text{-}EHH|_w = 3.14$

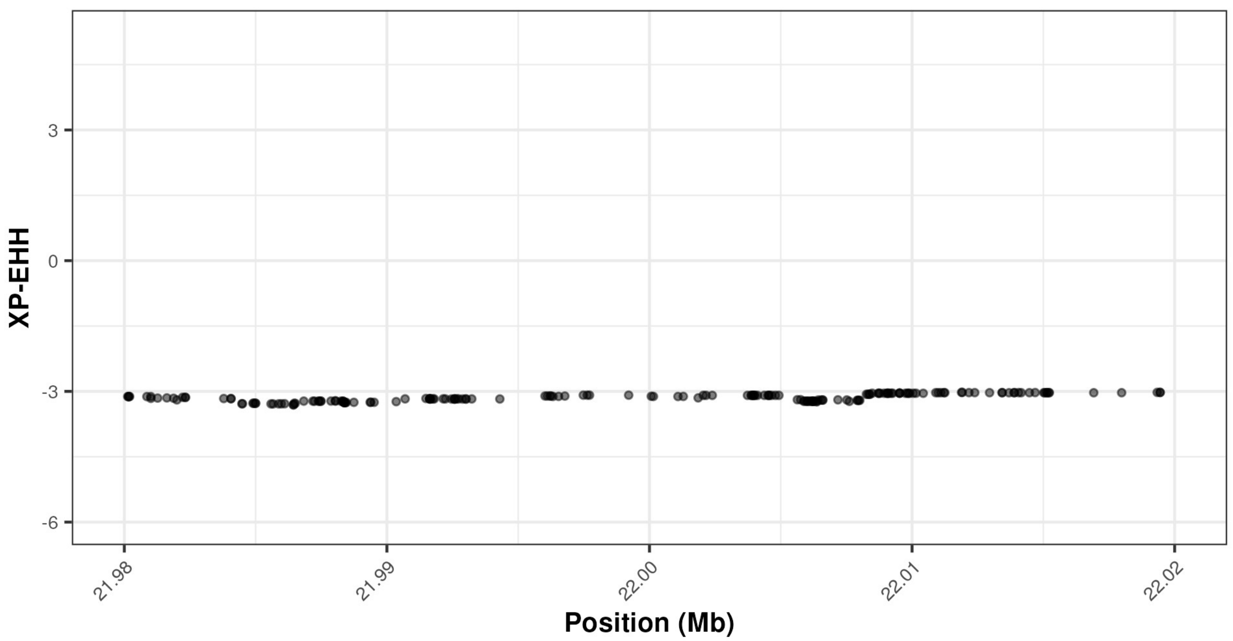

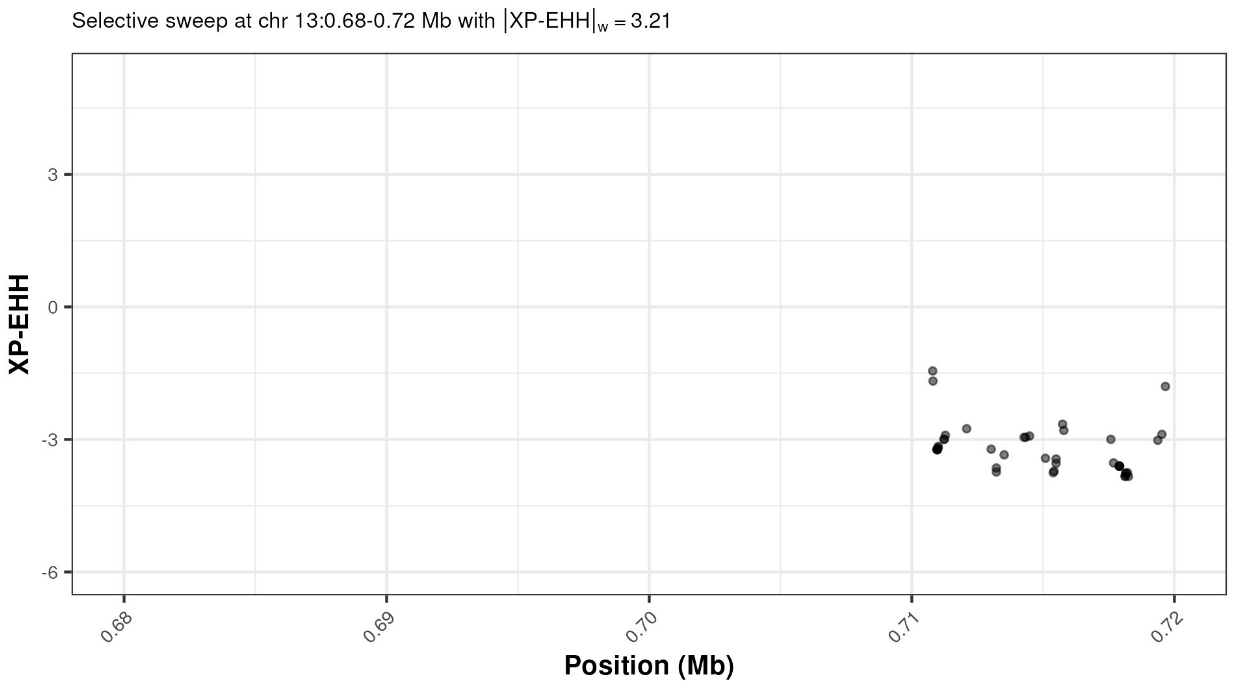
Selective sweep at chr 13:0.68-0.72 Mb with |XP-EHH|w = 3.21
XP-EHH
3
0
-3
-6
0.68
0.69
0.70
0.71
0.72
Position (Mb)

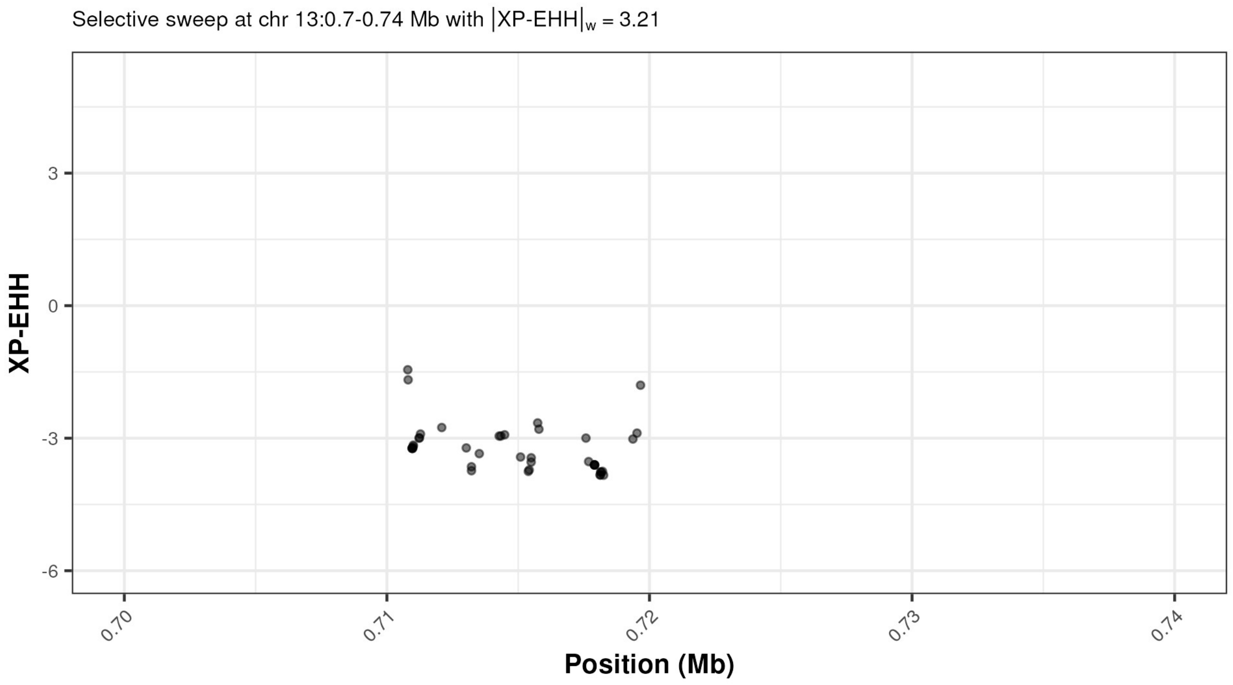
Selective sweep at chr 13:0.7-0.74 Mb with |XP-EHH|w = 3.21
XP-EHH
3
0
-3
-6
0.70
0.71
0.72
0.73
0.74
Position (Mb)

Selective sweep at chr 32:7.4-7.44 Mb with $|XP\text{-}EHH|_w = 3.38$

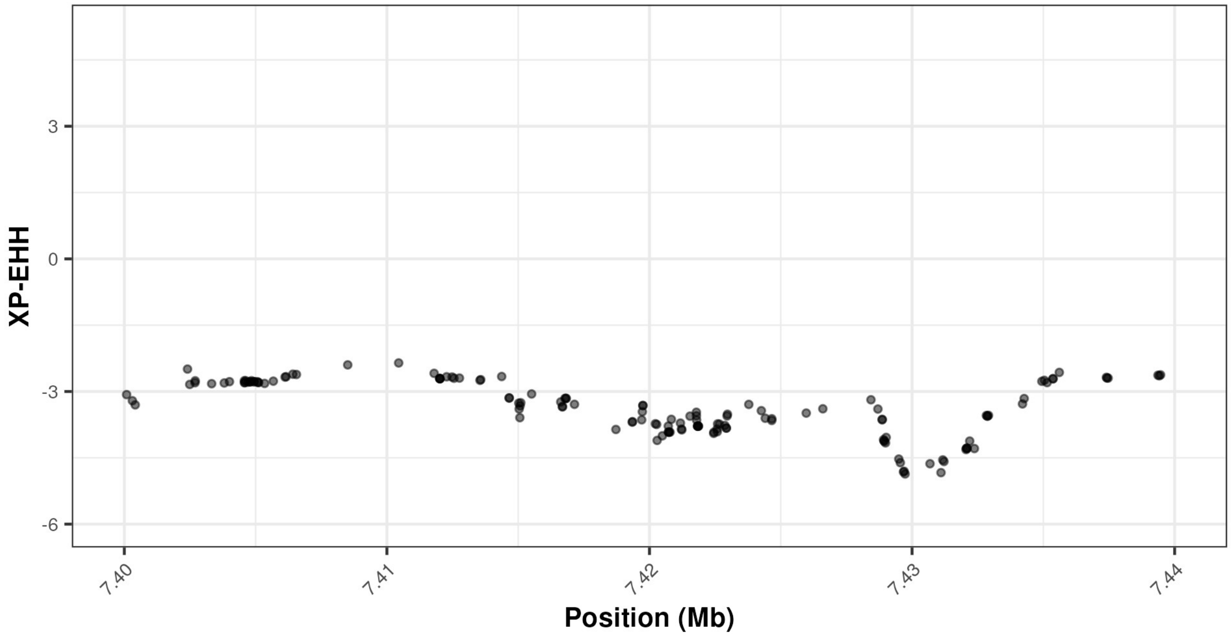

Supplement: Supplementary file 1 — Additional file 1: Figure S1. Principal component analysis of chicken populations. The first five principal components are shown, explaining 7.94 %, 5.35 %, 4.74 %, 4.54 %, and 3.99 % of the total genotypic variance. Figure S2. Heatmap of chromosome-wise fixation index (weighted $F_{ST}$) estimations between chicken populations. Figure S3. Cross-validation error for ADMIXTURE runs with increasing number of clusters (k). The lowest cross-validation error is reached at $k = 6$. Figure S4. Heatmap of chromosome-wise nucleotide diversity ($\pi$) estimations within chicken populations. Figure S5. The strongest signatures of selection according to $|XP-EHH|_w$ between the Ixworth chicken population and (a) commercial broiler line A, (b) commercial broiler line B, (c) commercial brown layers, (d) commercial white layers, and (e) red junglefowl. Strong selective sweeps are indicated by the red lines according to the top 0.1 %: (a) [file 12864_2026_12732_MOESM1_ESM.zip › Supplementary_File_S6.pdf]
